# Learning from the past: A reverberation of past errors in the cerebellar climbing fiber signal

**DOI:** 10.1371/journal.pbio.2004344

**Published:** 2018-08-01

**Authors:** Marc Junker, Dominik Endres, Zong Peng Sun, Peter W. Dicke, Martin Giese, Peter Thier

**Affiliations:** 1 Department of Cognitive Neurology, Hertie Institute for Clinical Brain Research, University of Tübingen, Tübingen, Germany; 2 Section on Computational Sensomotorics, Department of Cognitive Neurology, Hertie Institute for Clinical Brain Research, University of Tübingen, Tübingen, Germany; University of Minnesota, United States of America

## Abstract

The cerebellum allows us to rapidly adjust motor behavior to the needs of the situation. It is commonly assumed that cerebellum-based motor learning is guided by the difference between the desired and the actual behavior, i.e., by error information. Not only immediate but also future behavior will benefit from an error because it induces lasting changes of parallel fiber synapses on Purkinje cells (PCs), whose output mediates the behavioral adjustments. Olivary climbing fibers, likewise connecting with PCs, are thought to transport information on instant errors needed for the synaptic modification yet not to contribute to error memory. Here, we report work on monkeys tested in a saccadic learning paradigm that challenges this concept. We demonstrate not only a clear complex spikes (CS) signature of the error at the time of its occurrence but also a reverberation of this signature much later, before a new manifestation of the behavior, suitable to improve it.

## Author summary

The cerebellum allows us to rapidly adjust motor behavior to the needs of the situation. This cerebellum-based motor learning is guided by the difference between the desired and the actual behavior, i.e., by error information. It is usually assumed that not only immediate but also future motor behavior will benefit from error information because it induces lasting modifications of parallel fiber synapses on cerebellar Purkinje cells (PCs). These modifications affect the transformation of movement-related input into PC output and thence behavioral adjustments. Olivary climbing fibers, likewise connecting with PCs, are thought to transport information on instant errors needed for the synaptic modification yet not to contribute to error memory. Here, we report work on monkeys’ motor learning that challenges this concept. We not only identify a neural signature of the error at the time of its occurrence but also a reverberation of this signature much later, before a new manifestation of the behavior and therefore potentially involved in its improvement.

## Introduction

Ever since the publication of David Marr’s seminal theory of cerebellar cortex [1], thinking about the role of the cerebellum has revolved around the idea of motor learning, a view that has received support from studies of a variety of motor and oculomotor model systems [2–6]. A central feature of this concept has been the assumption that information on possible insufficiencies of the subject’s behavior, needed to improve future manifestations of the same behavior, is conveyed by one of the two types of afferent fibers reaching cerebellar cortex, namely climbing fibers, originating from the inferior olive. The second one is the mossy fiber system that conveys information relevant for the shaping of the behavior at stake, handed over to parallel fibers, which in turn connect with Purkinje cells (PCs). Climbing fibers contact PCs directly, where they ignite complex spikes (CS), usually studied as proxy for climbing fiber activity. According to the Marr–Albus–Ito (MAI) theory [1,7,8], the occurrence of an error changes the climbing fiber/CS discharge, which in turn induces heterosynaptic modification of simultaneously active parallel fiber synapses on the PC dendritic tree. It is this modification of the parallel fiber impact on PCs that in turn leads to changes of the PC output, which is ultimately responsible for the change of behavior. In other words, the MAI theory suggests that the climbing fiber system uses error information to facilitate a specific pattern of parallel fiber input, allowing the optimization of behavior. A central aspect of this concept is the assumption that the role of the climbing fiber signal is limited to providing information on the occurrence of an instant error. However, in order to avoid the same error in the future, the system should certainly conserve a memory of this error and consider it also for the shaping of future manifestations of the same behavior. This requirement is met by the MAI theory, as it posits longer-lasting changes of the synapses of parallel fiber with PCs brought about by the original error. In other words, a memory of past errors is implicitly stored in the parallel fiber synapses impinging on the PC.

Although this concept has received support from a number of studies, not all findings have been readily compatible with the idea that the role of the climbing fiber is confined to communicating instant errors. One example is our recent work on short-term saccadic adaptation (STSA), studied as a model of cerebellum-based motor learning, in which we observed patterns of CS that were at odds with the assumptions of the MAI theory [9]. In studies of STSA, the subject is asked to make a saccade towards a peripheral visual target. Then, while the eyes move to the target, the target is shifted to a nearby position [10], a shift that is unnoticed because of saccadic suppression [11]. Since the subject is unaware of the target shift, the eyes arrive at where the target was expected to be, which is why a subsequent corrective saccade has to be added in order to acquire the target at its final location. If the target shifts are repeated time and again in a consistent manner, one typically observes that the metric of the primary saccade adapts such as to bring the eyes closer to the final position of the target. STSA is a form of motor learning that depends on the integrity of the oculomotor vermis (OMV) [12]. When recording CS of OMV PCs, we surprisingly observed a gradual buildup of a specific saccade-related CS modulation that became most pronounced at the end of adaptation, i.e., at a time the error had been annulled, a modulation that actually outlasted the end of the adaptation period. On the other hand, we could not retrieve a significant influence of the original error on the CS discharge even early during adaptation [9]. Moreover, when studying a second form of oculomotor learning, likewise dependent on OMV PCs—smooth-pursuit adaptation—we made fully congruent observations, namely the absence of an error signature and the buildup of a stable CS pattern paralleling the learning [13]. Both paradigms have the drawback of inducing the buildup of strong behavioral changes that quickly reduce the original error, thereby potentially concealing its influence. This is why we decided to resort to a random error saccadic adaptation paradigm. It avoids both problems (the gradual buildup of strong behavioral changes and the accompanying diminution of the error), potentially obscuring the electrophysiological fingerprint of the original error. When analyzing CS of PCs in the OMV recorded in conjunction with the behavior, we found not only a CS signature of the error at the time of its occurrence but in addition a reverberation of this signature later, before a new manifestation of the behavior. Hence, the climbing fiber system helps to conserve information on error for the future, rather than being confined to conveying information on instant errors.

## Results

### Retinal errors induce trial-by-trial adaptation

**Fig 1:**
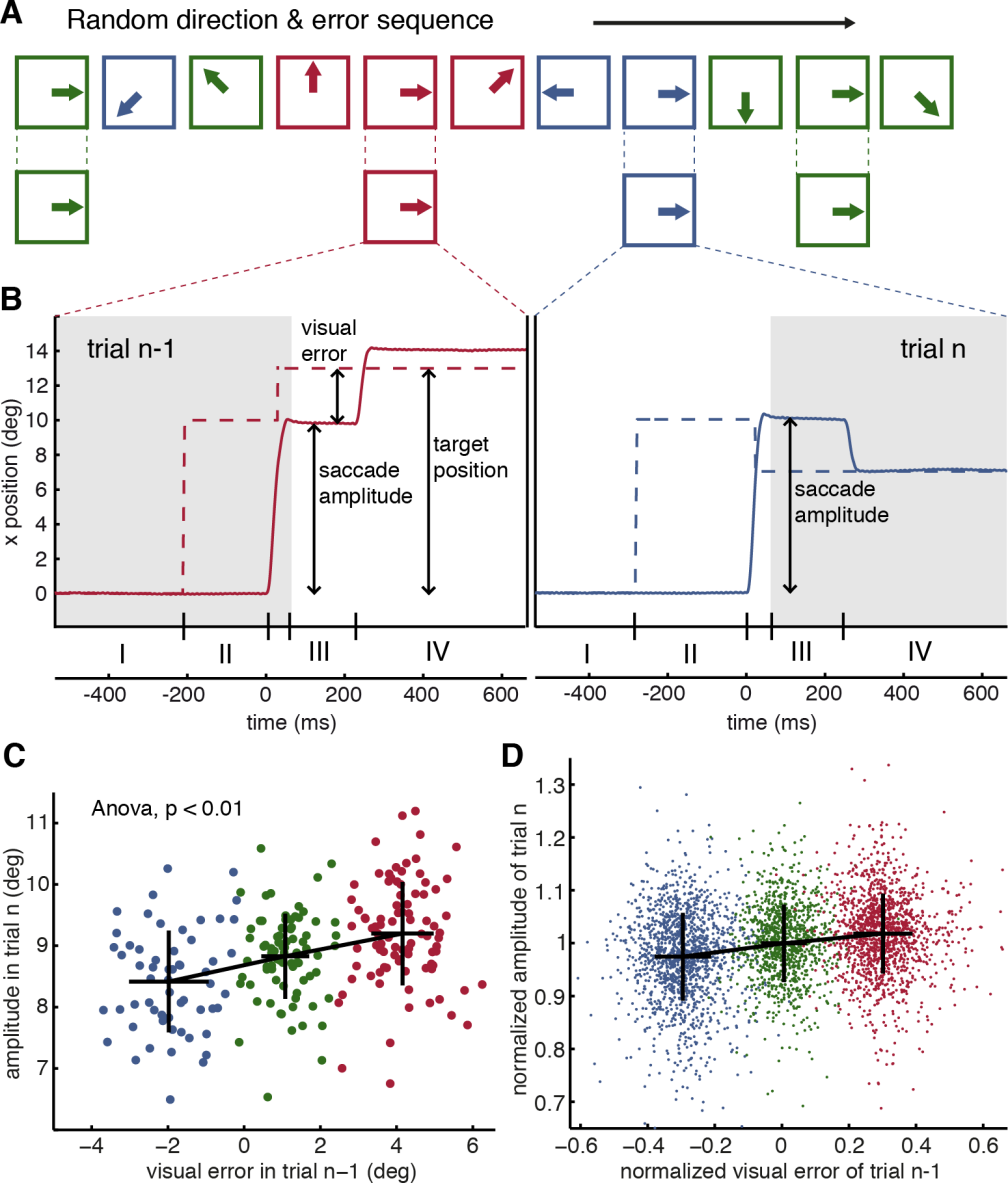
The random error paradigm and its influence on saccade amplitude. **A** Scheme explaining the selection of trials, considering the type of error and saccade direction in trial *n*. An arbitrary sequence of trials (first row), represented by boxes, is shown. The direction of the enclosed arrow indicates the direction of the primary saccade in the plane of the screen, and the color depicts the error condition of that particular trial (blue = inward error, green = no error, red = outward error). For the analysis of a particular direction, the sequence is filtered for trials of a specific direction (second row), and following trials are analyzed according to the error condition of the respective trial *n* − 1. **B** Trajectories of the visual target (dashed curves) and the changes in eye position they evoked (solid curves) in a sequence of two exemplary trials in which the target stepped out (trial *n* − 1, red traces) or in (trial *n*) during the primary saccade. The numbered intervals at the bottom mark the different periods considered for analysis: I, baseline fixation; II, primary visual error; III, secondary visual error; IV, post correction; the period of the primary saccade falls between intervals II and III. The nonshaded area marks those parts of the trials relevant for transferring error information from trial *n* − 1 to trial *n* for the purpose of adapting the primary saccade in trial *n*. Traces are aligned to the onset of the primary saccade. **C** Trial-by-trial adaptation in a particular direction (downwards). Data are taken from an exemplary session of monkey H. Saccade amplitude on trial *n* is plotted over the visual error in trial *n* − 1; highly negative visual errors correspond to an inward shift of the visual target on trial *n* − 1 (blue symbols), while highly positive visual errors correspond to an outward shift of the visual target on trial *n* − 1 (red symbols). The green symbols reflect trials without target shift. In this case, the resulting visual error reflects insufficiencies of the primary saccade. Crosses give the mean and standard deviation for each cluster. The means for the three conditions were significantly different (ANOVA, *p* < 0.01). **D** Normalized saccade amplitude as a function of normalized visual error. Population data is based on sessions from all three monkeys, considering only directions with a significant effect of trial-by-trial adaptation as described in the results section. Variables were normalized to the median of each data cluster—in terms of amplitude as well as visual error—obtained from individual sessions for the three target shift conditions before being pooled. The black crosses give the means and standard deviations for each cluster. This plot, generated for illustration purposes only, is based on a randomly chosen, representative subsample of 10% of the real data to maintain a clear visual separation of the three clusters. A regression yields a transfer of adaptation from the visual error in trial *n* − 1 to trial *n* of approximately 6% (slope of 0.059 ± 0.0046). Underlying data available from the Dryad Digital Repository: https://doi.org/10.5061/dryad.p88b8v8

The monkeys performed visually guided saccades in eight randomly chosen directions in the frontoparallel plane, and in each trial, there was a probability of one-third for the target to stay put, to jump partially back towards the center during the saccade, or to jump further out. As exemplified in Fig 1A and S1 Fig, as a consequence of intrasaccadic target jumps, the primary saccade over- or undershoots the target, respectively, causing a saccadic error that was subsequently corrected by a secondary saccade.

We investigated the effect that the retinal error in a certain direction in trial *n* − 1 had on the amplitude of the saccade in the next trial *n* made into the same direction. Note that as a consequence of the randomization, this trial *n* could directly follow trial *n* − 1 or be delayed by a varying number of in-between trials made into other directions (Fig 1A). Fig 1C plots the amplitudes of primary saccades in trial *n* as a function of the retinal error in trial *n* − 1 for a set of trials performed into a particular direction (downwards) for an exemplary individual session of one of the experimental animals. This plot clearly shows that a retinal error not only prompted a corrective saccade in trial *n* − 1 but also led to clear and significant (one-way ANOVA, *p* < 0.01) changes in the metrics of the saccade in trial *n*, increasing the amplitude of the primary saccade in the case of an outward error and, conversely, decreasing it in the case of inward errors. In other words, the amplitude changes observed in trial *n* were such as to reduce the size of a retinal error in trial *n* had it been subject to the same intrasaccadic target shift as trial *n* − 1.

The amplitude changes from trial *n* − 1 to *n*, reflecting trial-by-trial adaptation of saccade amplitude, reached significance in approximately 35% of all individual data sets (individual experimental sessions × eight directions) obtained from the three monkeys (one-way ANOVA with the repeated measure factor “error class,” *p* < 0.05 in 35.25% of the tests). When we considered only directions for which at least 90 trials both for trial *n* − 1 and trial *n* had been collected (30 trials for each error condition), the same analysis yielded significant amplitude changes in 50% (*p* < 0.05) of the cases. Fig 1D plots the pooled data of this analysis; the portions of directions with significant trial-by-trial adaptation for each direction and monkey can be seen in S2 Fig. Performing a regression in the normalized amplitudes of saccades in trials *n* (Fig 1D) resulted in a slope of 0.059 ± 0.0046, indicating that amplitude changes due to the visual error in trial *n* − 1 amounted to approximately 6% of the size of the error. The same regression analysis for trials from all three monkeys, considering directions with nonsignificant effect of trial-by-trial adaptation only, is shown in S3 Fig. The regression suggests a slight deviation from the horizontal in a direction that qualitatively corresponds to the one for significant trials in Fig 1D. The slope of 0.021 of this regression line is roughly three times smaller than the one for significant trials shown in Fig 1C and 1D.

The significant amount of transfer is all the more astonishing given the fact that, on average, trials *n* − 1 and *n* in a given direction were separated by a mean of 6.04 trials in other directions. We did not observe a consistent transfer of the visual error in trial *n* − 2 on trial *n* (slope of −0.0047; not significantly different from 0, S4 Fig). However, when again restricting the analysis to directions for which at least 90 trials for all three error conditions had been collected, 14.23% of the individual data sets yielded a significant correlation of the visual error in trial *n* − 2 and the amplitude in trial *n*, again indicating an amplitude change that would reduce the size of a persistent error.

### The probability of CS occurrence is influenced by visual error

**Fig 2:**
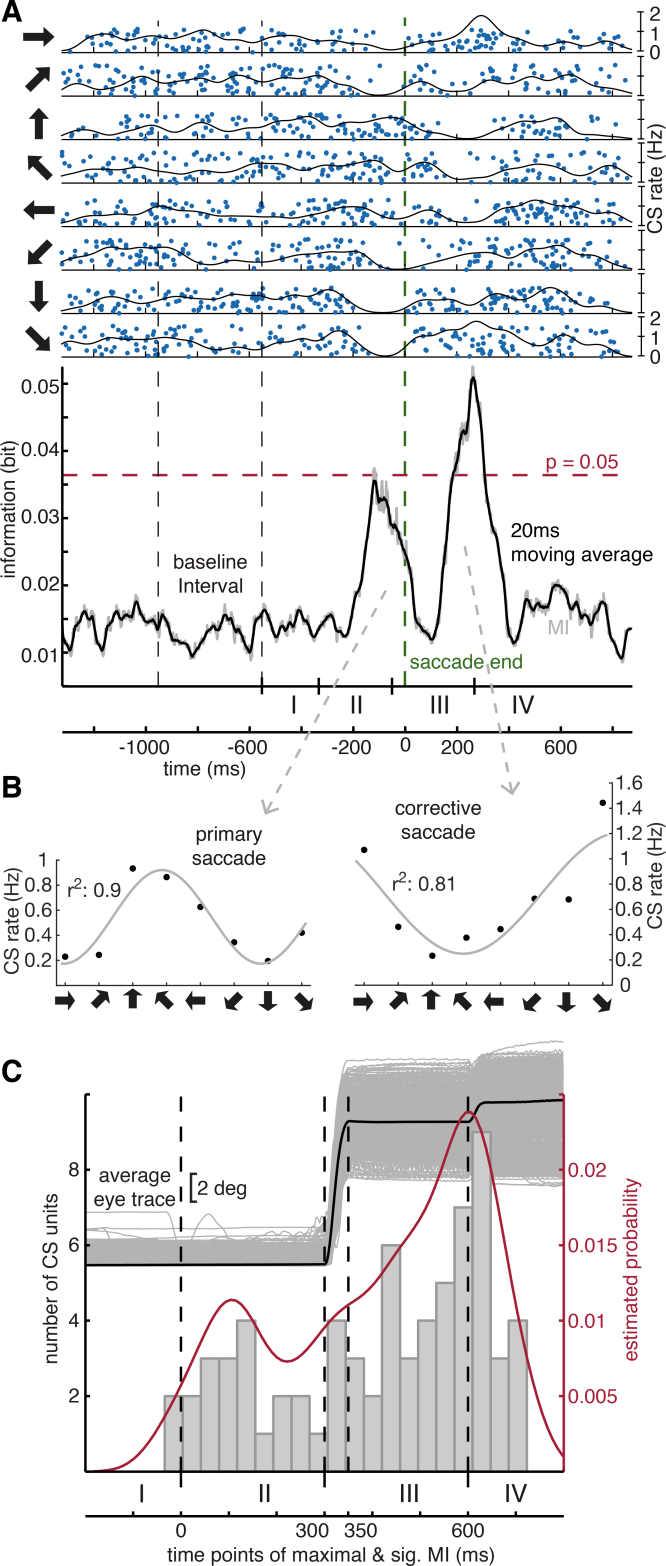
Single-cell and population analysis of direction-dependent CS modulation. **A** Exemplary CS unit. The upper panel is a raster plot of CS occurrence as function of time for the eight directions (arrows) tested. Only inward error trials are considered here. The two dashed vertical black lines demarcate the baseline fixation interval, on which the calculation of a significant MI threshold at *p* = 0.05 (red dashed line) rested upon. All trials were aligned to the end of the primary saccade, represented by the vertical dashed green line. The raster plots are complemented by CS density function (Gaussian kernel, width of 40 ms). The raw MI function (grey) was obtained by calculating the MI in a 250-ms sliding window and then smoothed with a 20-ms running average filter (black). Note that the inward error condition shown in this example induces a corrective saccade in a direction opposite to that of the primary saccade. Hence, the directions of visual errors prevailing in the periods before the primary and the secondary saccade respectively are opposite. Further note that the intervals distinguished at the bottom are approximate, as the different events jitter from trial to trial due to the monkeys’ response times. **B** Direction-tuning plot of the average CS rate in the intervals of the two MI peaks (width at half-maximum of each peak). **C** Distribution of the times of MI value maxima within trials based on all CS units exhibiting significant MI, pooled across error conditions. Units exhibiting their maximal response later than 200 ms after the onset of a corrective saccade were not considered in this histogram. The red probability density function was derived from the histogram via a kernel density estimate with a normal kernel of a bandwidth of 50 ms. For CS units with a directional preference, it illustrates how likely it is to observe its modulation at a certain point in time; it is intended to serve as a visual illustration aid. The black curve is the average eye trace (original eye traces in grey) and should help to relate the time course of the histogram to the main events of a trial (dashed lines from left to right: primary target jump; primary saccade onset; primary saccade offset; corrective saccade onset). Refer to S6B Fig for a description of how the time points indicating major events in a trial were calculated. Underlying data available from the Dryad Digital Repository: https://doi.org/10.5061/dryad.p88b8v8. CS, complex spikes; MI, mutual information.

The primary and the secondary error interval (Fig 1A) comprise periods in which the retinal target image does not fall onto the fovea, a “visual error” that is given by the vector connecting the fovea with the retinal target image. Even casual visual inspection of the 129 CS units collected in 110 sessions suggested that in these periods, the firing of many CS units seemed to deviate from baseline, at least for particular directions. In order to determine if the change in CS discharge provided information on the direction of the saccade within these periods or beyond, we calculated the mutual information (MI) between CS spike firing and trial direction over the time course of a whole trial (Fig 2A). This analysis was carried out on all 129 CS units and for each of the three conditions (inward error, outward error, no error control) separately. The results are summarized in S1 Table. We found that 79 out of 129 units exhibited a CS discharge modulation that resulted in at least one or more significant MI peak(s). To begin with, we determined the period in which the maximally significant MI peak was located, ignoring any possible secondary significant peaks that were seen in 23 CS units (Fig 2A). More than two significant peaks were not observed. Moreover, if a particular CS unit exhibited a significant MI peak in more than one condition (control, outward or inward error), only the condition with the maximal peak was considered (see S5 Fig for the full set of MI curves for the exemplary CS unit in Fig 2). Most (34 out of 79 CS units; 43.04%) of the maximal peaks were found in the secondary error interval (III) between the end of the primary saccade and the beginning of the corrective saccade. Significant MI in this period could reflect information on the direction of the preceding primary saccade as well as information on the direction of the intrasaccadic target shift. The second most (29 out of 79 CS units; 36.7%) preferred period was the primary error interval (II), the time between the onset of the saccade target and the end of the primary saccade. The remaining 16 units (20.3%) had their maximal significant MI peaks in the post-correction interval (IV) after the corrective saccade. The number of CS units with a significant peak in the MI was larger for the outward error condition than for the inward error or the control condition, respectively (49.4% versus 34.6% versus 16.5%, S1 Table). In a nutshell, almost two-thirds of all CS units provided an indication of directional selectivity.

We next mapped the time points at which the recorded CS exhibited their maximal MI into a histogram of a trial with normalized time course, eliminating differences in the duration of the various components of the sequence of events in a trial (for details, see S6B Fig), independent of whether the trial had been a control, an inward error, or an outward error trial. The resulting histogram, depicted in Fig 2C, shows two peaks, the larger one in the secondary error and post-correction interval (III–IV) and the smaller one in the primary error interval (II). The early, smaller peak represents information early enough to contribute to the decision on the direction of the primary saccade. The later, larger peak occurs about the time of the corrective saccade (however, in most cases, probably too late to contribute to its control). The notion that this second larger peak cannot be fully related to the control of the secondary saccade is actually supported by the fact that not all units contributing to it exhibited their maximal MI under error conditions: Actually, four of the 34 (11.8%) CS units (contributing to the later peak) were from the non-error control condition, 22 (64.7%) from the outward error, and eight (23.5%) from the inward error condition. In fact, 63.3% of all CS units exhibited their highest MI peak when aligned with points in time prior to the start of the corrective saccade, which further suggests that the secondary saccade itself is not the driver of this clear accumulation of MI modulation. Only 36.7% showed a maximal MI peak when the onset of the corrective saccade was chosen as reference for the alignment, 19% when it was the initial target jump, 25.3% when set to the start of the primary saccade, and, finally, 19% when the end of the primary saccade served as reference (S1 Table).

### The probability of CS occurrence reflects a memory of the past

We next asked if the occurrence of a specific visual error in trials *n* − 1 led to changes in the probability of CS firing in subsequent trials *n* in the same direction. To this end, we sorted trials *n* according to the presence of an inward error, an outward error, or the absence of an error in preceding trials *n* − 1. We then calculated the MI between the inward error and the control group, the control and the outward error group, and the inward error and outward error group and determined the maximally significant MI for each direction. Once we found that a CS unit exhibited a significant MI modulation for any of the three pairs, we regarded that CS unit as conveying reverberation of the direction of error in the previous trial *n* − 1.

**Fig 3:**
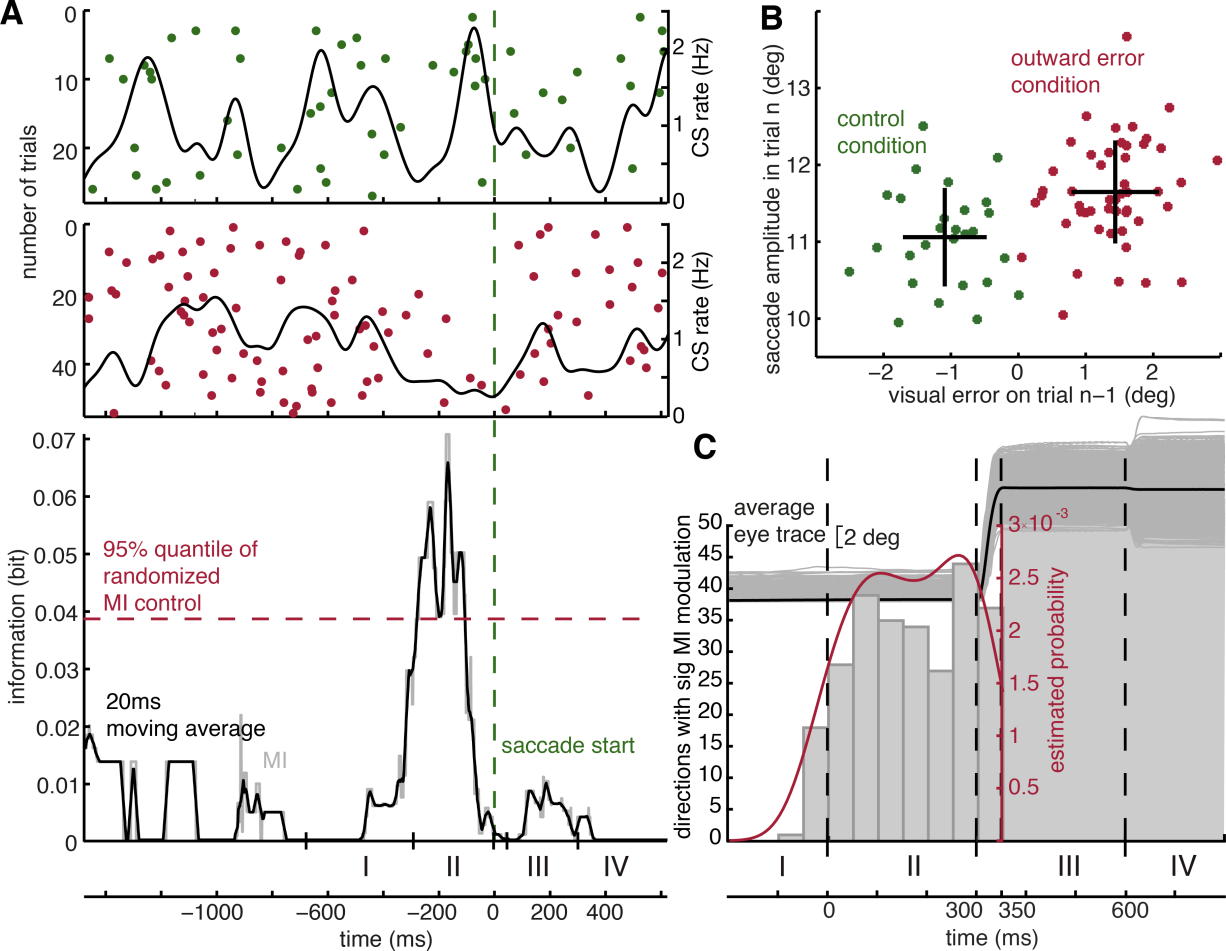
CS modulation is impacted by information from the past trial. **A** Exemplary CS unit represented by raster plot and the respective CS density curve (top) and plot of MI as function of time (bottom). The colors distinguish the error type in trial *n* − 1, preceding the trials shown in the raster plot (green: control, red: outward error); same direction for primary saccades. All trials were aligned to the onset of the primary saccade, represented by the vertical dashed green line. Note that the biggest difference between the two conditions lies right before the primary saccade, indicating that information about the condition in trial *n* − 1 is available before the primary saccade on trial *n* is executed. Note further that the inequality in the number of trials per condition is taken into account by the MI analysis. Further note that the designated intervals at the bottom are approximate and for visual illustration only, as the different events jitter from trial to trial due to the monkeys’ varying response times. **B** Plot of saccade amplitude in trial *n* as function of visual error in the preceding trial *n* − 1, demonstrating a clear impact of random errors on amplitude in one direction. The plot is based on the same set of trials from one direction of an exemplary session for which the CS discharge is shown in Fig 3A. Green dots correspond to trials with no target jump (control condition) in trial *n* − 1, and red dots indicate trials with an outward error condition (outward target jump) in trial *n* − 1. Black crosses indicate mean and standard deviation of each cluster. The mean saccade amplitudes are significantly different for the two sets (ANOVA, *p* < 0.01). **C** Distribution of times of significant trial-by-trial MI modulation of CS units. The time points of maximal significant modulation were mapped into the frame of a standardized saccade trial as in Fig 2C. The resulting distribution analogous to Fig 2C demonstrates that given a CS unit exhibits a change in CS activity in trial *n* due to a certain error condition in trial *n* − 1, it is most likely to observe this modulation before the primary saccade in trial *n*. The grey shaded area marks those intervals of trial *n* in which an influence of error information from trial *n* − 1 can no longer be disentangled from the error information associated with the performance in trial *n*. Underlying data available from the Dryad Digital Repository: https://doi.org/10.5061/dryad.p88b8v8. CS, complex spikes; MI, mutual information.

**Fig 4:**
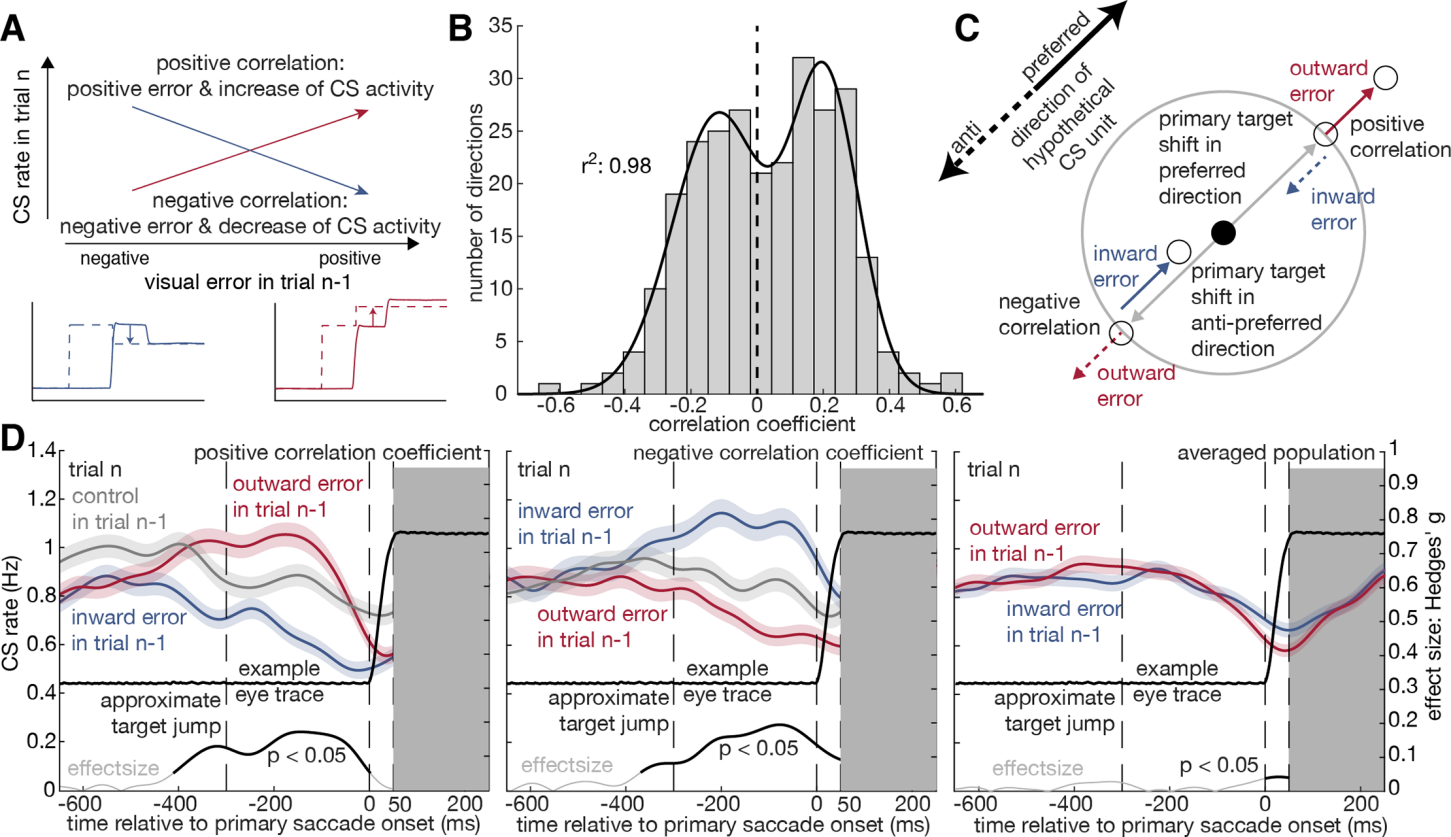
Correlation of visual error in trial *n* − 1 and CS activity in trial *n*. Correlation analysis of influence of the size of visual error in trial *n* − 1 on the average CS activity during the significant MI interval in interval II (the primary error period) of trial *n*. The correlation was carried out for all directions with significant MI. **A** Schematic graph explaining the emergence of positive and negative correlation coefficients between the secondary visual error in trial *n* − 1 and the CS activity in trial *n*. **B** The distribution of correlation coefficients obtained is bimodal and can be fitted by a sum of two Gaussians (r^2^ = 0.98). **C** CS units whose preferred directions are aligned with the outward error will exhibit a positive correlation with the size of the outward error and a negative correlation with the size of the inward error, the latter assuming that the nonpreferred direction is opposite to the preferred direction (left). Conversely, CS units whose preferred directions are aligned with the inward error will exhibit the inverse behavior (right). The colored arrows indicate the direction of the error (and the corrective saccade), the solid black arrow the CS unit’s preferred direction, and the dashed black arrow its nonpreferred direction. **D** Blue, grey, and red curves show the average CS spike density functions ± SEM prior to primary saccade onset in trial *n* for the three different error conditions (outward, inward, no error = “control”) in trial *n* − 1. The curve on the bottom shows the Hedges’ g effect size in grey and indicates a significant (*t* test, *p* = 0.05) difference between the red and the blue curve in black. The vertical dashed lines indicate from left to right the approximate time of the target jump, the start of the primary saccade as alignment point, and the approximate end of the primary saccade at 50 ms. The solid black curve in the center of each panel is an eye trace of a 10-degree saccade shown to facilitate relating the CS SDF to the time course of a saccade. For the rightmost panel, we skipped the averaged control condition to focus on the subtle difference between inward and outward error conditions. Note also that the control condition will be influenced by visual errors that might have prompted corrective saccades, as saccades amplitude vary across an ideal size and may even—depending on the individual—exhibit systematic deviations in the one or the other direction. Underlying data available from the Dryad Digital Repository: https://doi.org/10.5061/dryad.p88b8v8. CS, complex spikes; MI, mutual information; SDF, spike density function.

In total, 111 out of 129 CS units showed at least one significant MI peak in at least one direction. In an attempt to pinpoint the period exhibiting the maximal influence of past errors on CS firing, we resorted to the approach also used in the analysis of direction selectivity, namely to map the times of maximal MI modulation into a normalized saccade trial. The resulting population histogram (Fig 3C) shows a clear accumulation of information right before the primary saccade on trial *n* (S7 Fig summarizes the associated adaptation effects; see Fig 3A and 3B for an exemplary CS unit and the related behavior). Note that the MI modulation can be undoubtedly related to the error condition in trial *n* − 1 only until the end of the primary saccade. Beyond this point in time, any error information could be biased by the secondary retinal error of trial *n*. In other words, a population of 111 CS units (86%; 111 out of 129) offers information on the error in trials *n* − 1 in a period of time optimally suited to cause the observed changes of the metrics of the upcoming saccade in trials *n* due to preceding errors (see Fig 4D).

The MI analysis detects significant changes in CS firing but does not reveal if this change is a consequence of a drop in CS firing rates or, alternatively, an increase. In the case of saccadic adaptation prompted by error information that is consistent over long series of trials, it is the direction of the change that decides whether saccade amplitude is up- or down-regulated [9]. In order to clarify if the pattern of CS changed around the time of a saccade whose amplitude would change because of a preceding error, we calculated correlations between the size of the visual error in trial *n* − 1 and the mean CS rate in the time of significant modulation in interval II (the primary error period) of trial *n* (Fig 4). The distribution of correlation coefficients obtained based on pooling all directions from all CS units with significant MI (Fig 4B) is bimodal and can be fitted by a sum of two Gaussians (r^2^ = 0.98). Bimodality is to be expected, as CS units whose preferred directions are aligned with the outward error will exhibit a positive correlation with the size of the outward error and a negative correlation with the size of the inward error, assuming that the nonpreferred direction is opposite to the preferred direction. Conversely, CS units whose preferred directions are aligned with the inward error will exhibit the inverse behavior (see Fig 4A and 4C for illustration). This is the reason we distinguished the two pools of CS units with negative and positive correlation coefficients when calculating average CS density functions aligned with the onset of the primary saccade separately for the three error conditions (i.e., outward, inward, no error control) (Fig 4D). As can be seen there, the average CS density functions clearly diverge before saccade onset. In the case of positive correlation coefficients (i.e., CS unit’s preferred direction and direction of outward error parallel; Fig 4D, left panel) the CS density function exhibits more activity in the period of interest than the control function and the one for inward errors. In the case of negative correlations (middle panel), the order is reversed. Finally, pooling CS units with positive and negative correlation coefficients annihilates the differences (right panel).

Taking the differences in CS activity one step further, one would expect that the above shown effects of adaptation also find a direct reflection in the behavior, namely the saccade amplitude in trial *n*. By applying the same segregation into error directions in parallel and antiparallel to the preferred direction of a CS unit, we calculated the correlation between CS activity in the same interval—prior to the primary saccade—of trial *n* as in Fig 4B and the amplitude of the primary saccade in trial *n*, which is shown to undergo adaptation on the population level (S7 Fig). As a result, we see a shift between the populations indicating direct alteration of the saccade amplitude by the occurrence of CS in the primary error interval (S8 Fig)

Links between the firing of simple spikes (SS) and CS in the context of motor learning are well established [3,14,15], which is why we wondered if the observed effects on CS might actually be secondary to error-related changes in simple spike discharge. In order to address this concern, we performed an MI analysis on the SS discharge for all units and directions for which we had obtained a significant effect of past errors on the CS, for which well-isolated SS responses were available (82 units). In other words, we checked if those datasets, which exhibited a significant modulation of their CS, also exhibited a significant modulation of their SS. The analysis was modified to consider SS within a 150-ms window and not a 250-ms window as in the case of the CS analysis. We found a significant modulation of SS responses for only 35 directions, which are only 18.1% of the 193 directions with a significant MI modulation of CS activity (compare to Fig 3C). Hence, although in very few cases, a role of the preceding SS activity for the CS modulation cannot be excluded; the analysis clearly speaks against the possibility that the SS would be able to account for the reflection of past errors in the CS activity. S9 Fig conveys the distribution of time points of significant SS modulations.

One might argue that the subtlety of the changes of the CS discharge contradicts the clear behavioral effects and may doubt that the altered probability of CS before a primary saccade influenced by past errors is actually relevant for the behavioral change. Continuing in the same vein, one may also wonder if the pattern of CS occurrences is really able to reveal the full role of the CS. This was the reason that we decided to also take a closer look at the duration of CS, expecting to see changes, possibly complementing changes in CS discharge. However, as described in S1 Text, we failed to unravel any changes in the duration of OMV CS. The analysis of CS duration was restricted to a group of 38 particularly well-isolated and stable CS with discharge rate dependencies not different from the complete sample in terms of directional preferences and an influence of the error (see S10 Fig, S11 Fig). Therefore, it is unlikely that a simple sampling bias can account for our failure to detect meaningful CS duration changes.

## Discussion

We studied single-trial saccadic adaptation induced by randomized visual errors. We found not only clear evidence of adaptation but also conspicuous accompanying changes of the probability of the occurrence of CS fired by PCs in the OMV, reflecting the influence of a visual error annulled by a subsequent saccade. In accordance with previous studies on visually guided hand movements [16,17], saccades [18,19], and smooth-pursuit eye movements [20,21], we observed that in many cases CS responses to visual errors were directional, i.e., they were sensitive to the direction of the vector connecting the fovea with the location of the target image in the periphery of the visual field. Interestingly, we found that sensitivity to a particular type of error direction depended on whether the error preceded the primary or the secondary saccade (corrective saccade) with many more CS units responsive to the latter. Only the secondary error is a performance error. Hence, the observed stronger influence of the secondary error on the CS firing might be expected in the light of the MAI theory, which posits a role of the climbing fiber system in reporting performance errors. A less-ambitious explanation may in principle be a lower sensitivity to the larger amplitudes characterizing the primary error. Although it is usually assumed that CS do not exhibit significant amplitude tuning [19], our data do not allow us to firmly exclude a role of saccade amplitude. In any case, the observation of directional sensitivity to the presence of a visual error is in accordance with the notion that the climbing fiber system offers information that may change future behavior such as to avoid the occurrence of similar errors [9,17,22]. How could changes in the probability of CS that coincide with the occurrence of an error change future behavior? The standard answer to this question, based on a solid body of evidence, is the assumption that these changes will induce longer lasting modifications of parallel fiber–PC synapses, modifying the transmission of future signals relevant for the behavior at stake [23]. Without questioning the viability of this mechanism, our observations suggest an alternative, not necessarily exclusive way past error experiences might shape future behavior, namely by changing the probability of future CS occurrences. Our analysis clearly showed that the presence of a visual error in the period following a primary targeting saccade, induced by shifting the target during the saccade to a new position, led to highly significant changes in the probability of CS preceding the primary saccade in subsequent trials made into the same direction.

Although unequivocal, the dependencies of the CS discharge on past and current visual errors were extremely subtle in absolute terms and could only be revealed with the help of sophisticated statistical approaches. This subtlety is of course not surprising given the notoriously low probability of CS occurrences. The analytical consequences of this hallmark of CS were further aggravated by the random structure of the adaptation paradigm used, which prevented simple averaging across sequences of stereotypical trials with consistent errors. The lack of consistency of error information notwithstanding, individual visual errors had a profound effect on primary saccades in subsequent trials into the same direction, although on average, approximately six trials into other directions were interleaved. Our observation that inconsistent individual errors have a clear effect on future manifestations of the same behavior is in accordance with previous work on visually guided reaching [24], saccades [25], and smooth-pursuit eye movements [20]. It is also in line with recent findings [26] that suggest that the effect of unexpected errors may actually be stronger than the effect of gradual error increase yet less stable.

We interpret the significant change in the probability of CS firing before the primary saccade in a trial made in the same direction as the one in which an error had occurred in an earlier trial as a reverberation of the influence of the past error. One might argue that it is not a reverberation of the past error that determines the CS modulation but the motor command driving the new primary saccade. We think that a consideration of the timing clearly speaks against a role of a motor command generated upstream of the cerebellum: the CS reverberation starts roughly around 300 ms before the upcoming primary saccade (Fig 4D). On the other hand, saccades evoked by microstimulating the deeper layers of the superior colliculus (SC), the likely source of the motor command, have latencies around 20–30 ms [27]. Even if we assume that a command signal from the SC may influence the inferior olive a few ms earlier than the overt onset of the saccade, it would be too late to cause the CS modulation. Actually, a much more plausible scenario, linking behavioral changes and the changes of the CS firing, is suggested by a consideration of the SS discharge. A fraction of the PCs tested showed significant changes in their SS around the time of the upcoming primary saccade (S12 Fig), either with or without accompanying CS reverberation activity prompted by past errors (S9 Fig). We know from previous work that saccadic learning is a consequence of changes of a SS population signal [28,29]. Against this backdrop, it seems likely that the SS changes observed in our experiment in trials in which the upcoming primary saccade is influenced by past errors in the same direction is responsible for the adaptation effects observed in such trials. In other words, the SS modulation most probably mediates the modification of the motor command needed. This modulation comes too late to account for the CS reverberation signal. Conversely, the temporal relationship of the CS and SS signals might be compatible with the former causing the latter, considering the well-established pauses of SS firing following CS, a consequence of the recruitment of inhibitory interneurons and the activation of calcium-dependent potassium channels of PCs [30,31]. On the other hand, the modulation of the SS discharge close to the primary saccade might also be a consequence of changes of the strength of parallel fiber–PC synapses brought about by the CS signal, reflecting the performance error in the earlier trial. Not surprisingly, these changes in parallel fiber efficacy would become detectable statistically only at times of sufficient mossy fiber drive, i.e., at the time of the new primary saccade. This latter scenario is intriguing, as it might offer a tentative answer to the question of how the CS reverberation signal might be generated in the first place: the idea is that performance error-related CS activity leads to changes of the PC SS population output, which is then fed back to the inferior olive by nucleo-olivary projection neurons in the deep cerebellar nuclei. Of course, we can only speculate as to how the putative feedback influence might lead to the delayed activation of the inferior olive, compatible with the timing of the CS reverberation signal.

In any case, although clear, the influences on the CS firing frequency are subtle. This is why we wondered if the changes of the pattern of CS occurrences due to past errors might be accompanied by changes in the duration of CS. The idea behind this interest in CS duration is that the amount of calcium influx, responsible for the CS impact [23,32], should not only increase with CS frequency but also with CS duration. As both seem to be linked [33], one might expect that subtle changes in CS frequency might become amplified by yoked changes in CS duration. Actually, the idea that changes in CS duration may contribute to learning-based behavioral changes has received support from a recent study of the role of floccular PC CS in smooth-pursuit eye movement adaptation [20]. However, we failed to unravel any changes in the duration of OMV CS. Notwithstanding the possibility of an additional role of the CS duration or, more generally, CS waveform, our inability to reveal it in our data contrasts with the statistically clear changes in the CS discharge rate, in particular the changes we found as a reflection of past errors in the CS discharge before the upcoming saccade.

Previous discussions of the role of the climbing fiber in saccadic learning have been characterized by seemingly incompatible findings. Soetedjo and colleagues [19,22] have emphasized the presence of an error signature in the ongoing CS firing of OMV PCs in the secondary error interval (III) before the corrective saccade, when using a double-step paradigm. On the other hand, Catz and colleagues [9], when resorting to saccadic adaptation paradigms, in which the target was consistently shifted outward or, conversely, inward during the saccade, inducing gain increase and decrease adaptation, respectively, failed to reveal an influence of the error-driving adaptation. However, they demonstrated the gradual buildup of a strong and highly specific CS modulation around the time of the primary saccade, paralleling the buildup of adaptation. This modulation reached its maximal expression, easily visible already in the raw raster plots, at the end of adaptation, when the error had been effectively annulled. Completely analogous observations were subsequently obtained by Dash and colleagues [13] when studying smooth-pursuit eye movement adaptation. The results presented here suggest how these seemingly incompatible results of Catz and colleagues and Dash and colleagues on the one hand and Soetedjo and colleagues on the other hand can be easily reconciled. We have demonstrated a subtle influence of error in the period before the secondary saccade, as well as a clear influence of this past error before the next primary saccade in the same direction. While the earlier influence is in line with the Soetedjo and colleagues results, the latter is in accordance with the observation of Catz and colleagues.

As sketched further up, we suggest that changes in the CS discharge due to a performance error cause changes in the SS population output, which is in turn able to influence the inferior olive, thereby reminding the inferior olive of a past error. At the level of the inferior olive, this input would in principle be able to interact with information on a new performance error if the timing of the two inputs could be matched. In this case, a new error, consistent with the old one, would boost the climbing fiber output. On the other hand, if the two were contradictory, they would annihilate the climbing fiber modulation. In other words, this mechanism would basically serve as an error integrator that would provide a persistent CS modulation able to stabilize the new behavior, reflecting consistent error reports even in the absence of fresh errors of the same kind. In other words, we think that our results fully support the notion that the CS serves a dual role, initiating new behavior and as well stabilizing it in the absence of countermanding error signals, a notion that is line with similar thoughts prompted by recent findings on the role of CS in eye blinking [34].

## Materials and methods

### Ethics statement

The project relied on nonhuman primates (NHPs; macaque monkeys) in its study of the cerebellar underpinnings of saccadic learning as the oculomotor systems of NHPs and of humans are virtually identically. A similar correspondence does not hold for any other group of mammals. Moreover, only NHPs offer the cognitive flexibility and trainability required by the demanding behavioral paradigms needed. There is no alternative to the NHP model in studies of the neuronal underpinnings of saccadic learning and the role of the cerebellum. Any effort was undertaken to keep the number of animals used in the work reported as small as possible and to minimize the burden for those involved in full accordance with the 3R principle. This also involves continuous efforts to refine the experimental procedures, e.g., by optimizing surgical protocols, by developing less invasive head holders largely integrated into healthy tissue, or by warranting group housing of experimental animals in large rooms accommodating completely normal social interactions. A complete description of all relevant aspects of the procedures and the legal and organizational framework is provided in the Materials and methods section.

The experiments were approved by the local authorities in charge (Regierungspräsidium Tübingen and Landratsamt Tübingen, license N1/08 and N6/13), conducted in accordance with German and European law and the Guidelines of the National Institutes of Health for the Care and Use of Laboratory Animals, and carefully monitored by the veterinary service of Tübingen University.

### Animals

Three male rhesus monkeys (*Macaca mulatta*) were prepared for high-precision eye position recording using the scleral search coil technique [35]. To painlessly immobilize the monkeys’ heads, a titanium pole was attached to the skull with titanium bone screws three to six months prior to the initial training. After a successful training period of approximately one to three months (dependent on individual progress), recording chambers for electrode access to the OMV (i.e., to lobule VIc/VIIa), also titanium-based, were implanted in the midline over the posterior part of the skull, tilted backward relative to the frontal plane by 30°. All surgical procedures were conducted under general anesthesia, and after surgery, monkeys were supplied with analgesics until full recovery. See Prsa and colleagues [36] for more details.

### Behavioral paradigm

We confronted the saccadic system with randomized visual errors by deploying the following paradigm: A trial started with the presentation of a fixation target (white dot, diameter 0.2°) straight ahead of the monkey on a monitor. After a variable fixation period of 1 to 1.2 seconds, the fixation target disappeared, and at the same time, a saccade target with the same shape and color became visible at an eccentricity of 10° in one out of eight randomly chosen directions (0°, 45°, etc.). During the ensuing “primary” saccade to this target, it was randomly stepped further out by 3° (“outward error” trials), back towards the fixation point by 3° (“inward error” trials), or stayed put at its original location (“control” trials). The probability of these three trial variants was one-third each. Note that in all three cases, the primary saccades were guided by the target at its original location, and the corrective saccades were either in the same (in the case of an “outward error” trial) or in the exact opposite (in the case of an “inward error” trial) direction of the primary saccade (see also Fig 1A, S1 Fig). Therefore, up to the end of the primary saccade, the experimental conditions were identical across the different error conditions for all trials performed into the same direction. Overall, the probability of having a trial in a specific direction with a specific target displacement was 1 out of 24. Different trial types were randomized in order to prevent the formation of an expectation of specific errors. In order to reveal the influence of a saccadic endpoint error in a given trial *n* − 1 in a particular direction on an upcoming trial *n* in the same direction, we first sorted all trials according to direction and then—for a given direction—according to the type of error prevailing in trial *n* − 1 (i.e., no error, inward error, outward error; see Fig 1). For later analyses, the visual error was defined as the target position minus the eye position, both determined 50 ms after the end of the primary saccade.

### Electrophysiological recordings from the OMV

Extracellular recordings were carried out with commercial glass-coated tungsten electrodes. Online signal processing, filtering, and template-based spike detection were done with commercial Alpha Omega hardware and software. The position of the region of interest, the OMV, was approached based on stereotactic calculations that considered the position and orientation of the chamber relative to the brain, validated by postsurgical anatomical MRI scans. The OMV could then be easily identified relying on its characteristic electrophysiological signature, the dense saccade-related background activity reflecting granule cell activity, and the high probability occurrence of well-isolated saccade-related Purkinje and Golgi cell action potentials. Moreover, we observed that the eye movement–related granule cell multiunit background was correlated with conspicuous saccade-related deflections in the local field potential (LFP; low pass-filtered raw signal with a cutoff frequency of 150 Hz). These saccade-related LFP deflections were not observed outside the OMV. Distinct LFP deflections emerged as a consequence of the occurrence of CS. Their polarity depended on the layer in which the CS were recorded (see S10A–S10C Fig). Individual CS were identified based on their long duration, their multiphasic structure with several individual spikelets, the prominent footprint in the LFP mentioned before, and, finally, their conspicuously low firing rate. Individual PC CS were taken as proxy of climbing fiber activity. Spikes with more “simple” morphology were taken as SS of PCs if the stream of these SS was interrupted by occasional CS, and correspondingly, CS-triggered SS histograms showed a SS pause right after the CS. SS and CS were detected online using template matching, and the validity of the online detection of CS was checked offline in the stored raw records sampled at 25 kHz. The offline visual inspection was supported by considering the CS stamps in the LFP mentioned earlier and suggestions made by an offline spike sorting algorithm (WaveClus2, written by [37]).

### CS duration and number of CS spikelets

The experiments involved 250 to 1,800 individual trials; in other words, they usually required fairly long recordings of individual CS units. Hence, not surprisingly, they were in many cases associated with changes in the signal-to-noise ratio (SNR) and changes in the morphology of recorded CS units, arguably a consequence of subtle shifts in the position of the electrode tip relative to the unit. Unlike the rather robust analysis of CS occurrences, any estimate of CS morphology can be expected to be influenced by these long-term changes in the quality of recordings and therefore to confound the detection of task-dependent effects. In an attempt to avoid this problem to the best possible extent, we restricted the analysis of duration and spikelet number to a subset of 38 CS units out of the total of 129 units recorded in which the SNR was particularly good and the appearance of spikes stable over the whole duration of the experiment.

Duration measurements and spikelet counts were based on visual inspection of individual CS by one of the authors (MJ). His judgment was doubled by another one, ZS, in one-third of the units. CS duration was measured from the first deflection of the extracellular potential up to the final return to baseline potential. To determine the number of spikelets within a CS, we counted the number of full oscillations following the initial deflection. The visual inspection of CS was carried out with the two investigators being blind as to trial type or time of CS occurrence relative to trial onset. Whereas the spikelet numbers estimated by the two authors did not differ, their estimates of CS durations deviated significantly (bootstrap test; *p* < 0.05 for the duration means; *p* > 0.05 for the number of spikelets). In other words, the recognition of spikelets had been consistent, but obviously, the subjective criterion for the end of a CS had been systematically different, with ZS reporting on average 2.4 ms shorter durations. However, this difference between observers was nondetrimental as it did not affect the comparison of CS durations across conditions.

We relied on the measurements of MJ and ZS for the analysis of dependencies of CS duration on visual error. We first determined the preferred direction of CS units relying on control trials (i.e., no intrasaccadic shift of the target). The preferred direction was obtained by identifying the direction with the highest probability of CS occurrence in the secondary error interval (interval III in Fig 1B), i.e., the period from primary saccade offset to corrective saccade onset considered to last for 250 ms, using a peri stimulus time histogram (PSTH) analysis. We relied on control trials for the identification of the preferred direction, which avoids a possible mixing of contradicting directional activity from around the primary saccade and the subsequent corrective saccade. Hence, subsequent analyses of dependencies of CS duration were restricted to the preferred direction and to trials with an outward error in trial *n* − 1, as they have the advantage that—in contrast to inward error trials—both the primary error (i.e., the retinal vector pointing to the peripheral target before the generation of the primary saccade) and the secondary error (i.e., the retinal error that results from the target shift during the primary saccade) are in the same direction. As we could corroborate the tight relationship between CS duration and the number of spikelets (see S10D Fig) reported by previous work [20,33], we could confine the analysis of possible learning-related changes of the two CS parameters to CS duration.

For the analysis of learning-based changes in saccade amplitude, we compared the amplitude of the primary saccade in a given trial *n* with the one in the last preceding trial *n* − 1, having the same direction. Note that due to the random structure of the paradigm, the time between the two members of this pair varied depending on the number of intervening trials made into other directions. One trial lasted for 2.2 s and was followed by an intertrial interval of 0.1-s duration. In order to investigate the relationship between behavioral changes and changes in CS duration, also used as proxy of the number of CS spikelets, we extracted all pairs with CS in the secondary error interval (III) and divided the pairs up into three groups, containing *n* − 1 trials with short-, medium-, or long-duration CS. The differentiation of the three duration classes was based on first calculating the mean CS duration plus standard deviation for each unit and then to decide if the duration of an individual CS would fall into the middle range of the population distribution (mean ± 0.44 std ≙ medium duration), below (≙ short duration), or above (≙ long duration) this range. S11A Fig compares the resulting distributions of short-, medium-, and long-duration CS. For details, compare Yang and Lisberger [20].

### MI analysis of CS firing

To determine whether the occurrence of CS carries information about saccadic errors, we measured the MI between the CS discharge Y and a behavioral variable of interest X in 250-ms sliding windows, a window width chosen to approximate the mean CS rate, thereby ensuring a maximal temporal resolution without the risk of spurious transients. For a CS unit firing on average at 1/s, a 250-ms window will contain between 0–2 CS with a probability of approximately 99% at an entropy of 0.89 bit, which is therefore the upper limit for the MI in our analysis and would allow us to distinguish approximately between two saccade directions. Since the actually observed MI values are much lower, increasing the window size would only reduce temporal resolution, with little extra gain in MI resolution. This would make it difficult to assign MI peaks to particular trial phases. The reason for the 20-ms smoothing kernel was our interest to make the curve more easily visually interpretable by smoothing it. The value chosen is admittedly arbitrary, yet none of our quantitative results depend on this choice. MI was given by:

$$MI(X;Y)=\sum_{x}\sum_{y}p\left(x,y\right)\;log\;(\frac{p(x,y)}{p(x)p(y)})$$

where the joint distribution p(X,Y) = p(Y|X)p(X) factorizes into the marginal distribution of the behavioral variable, p(X) and the conditional distribution of the CS discharge given the behavior p(Y|X). We control p(X) by choosing the stimuli, estimate p(X|Y) from the data, and compute *p*(*Y*) = ∑_*x*_*p*(*Y*,*x*) by marginalization. To reduce the probability that any result obtained might be due to random fluctuations of the very low complex spike rates (approximately equal to 0.5–2 Hz), we computed a baseline distribution of MI in the fixation interval (interval I in Fig 1B), which—by construction of the paradigm—could not contain any saccadic error-related information. We tested the response in every 250-ms window against this baseline distribution to determine whether it was likely to contain an amount of MI significantly above baseline. Hence, the MI value that had to be exceeded to reach significance at *p* < 0.05 depended on the individual unit.

We chose MI for two reasons: first, it is a very general, nonlinear measure of dependency between two random variables, which relates to other widely used measures like correlation or classification rate under specific distributional assumptions [38]. Compared to these measures, MI has the (theoretical) advantage of being invariant against information-preserving transformations of the random variables, such as relabeling of the error directions or nonlinear one-to-one transformations of the spike rate, thereby obviating the need to build an explicit decoding model. Secondly, it was employed in previous studies on the role of complex spikes in saccadic error encoding [9,17,19]. Thus, we maintain comparability to these studies.

Measuring mutual information from neural data is a notoriously difficult problem, typically due to an overestimation bias, which results from small sample sizes [39]. Several approaches to deal with this problem were proposed in the past. For instance, Panzeri and Treves [40] compute an analytical expansion of the bias and subtract the leading terms from the mutual information estimates. In Nemenman and colleagues [41], a Bayesian approach is derived, in which the authors construct a flat prior on the mutual information in the limit of a very large response space. We resorted to the Bayesian binning approach [42] developed by one of the authors (DE), which solves the overestimation problem by Bayesian model selection: a prominent source of mutual information overestimation are noisy parameter estimates. Bayesian binning gives high posterior weight to models whose degrees of freedom are well constrained by the data, thus effectively reducing this bias. It was demonstrated [42,43] that this approach works well on extracellular single-cell recording data. Mutual information analysis was coded in C++ and interfaced by customized Python and Matlab scripts. A detailed description of the Bayesian binning algorithm can be found in [42]. All non-information-theoretic analyses were performed with customized Matlab scripts.

To identify direction selective CS units, we grouped trials according to the direction in which the visually guided saccade was performed and estimated the mutual information between the occurrence of a CS (spike count) and the direction it was observed in (behavioral variable). This allowed us to directly determine if a CS unit is selective for saccade direction and when this selectivity occurs in the time course of a trial (Fig 2A).

We also calculated the MI between the error type in trial *n* − 1 (behavioral variable: no error (control trials), inward error trials, outward error trials) and the occurrence of CS (spike count) in subsequent trials *n* in the same direction (Figs 1B and 3A) for each direction independently.

We furthermore explored the effects of aligning the spike train data in four different ways prior to the MI analyses, namely with respect to the primary target jump, the start of the primary saccade, the end of the primary saccade, and the start of the secondary, corrective saccade (the latter of course does not exist in the case of control trials, i.e., trials without secondary target shift). Hence, for every recorded CS unit we obtained 11 MI time courses (three error categories × four alignments—one missing alignment in case of control trials) for each direction. An example of why an individual alignment for each CS unit is needed can be seen in S6A Fig. It shows MI curves for the outward error condition aligned either to primary saccade onset (left) or to the onset of the corrective saccade (right). Only in the case of the latter is a significant MI peak at the time of the corrective saccade is visible.

We regarded a CS unit as significantly modulated if its MI curve crossed the significance threshold of *p* < 0.05 in at least one of these 11 possible combinations of category and alignment. To determine this threshold, we estimated the distribution of the MI in a baseline interval comprising 400 ms of fixation, 200 ms prior to the initial visual target jump for each combination of error category and alignment from the MI profile. Since we computed MI in 250-ms sliding windows, we corrected the individual estimates by assuming that they are correlated because of the overlaps between these sliding windows. We assumed that the MI estimates within the sliding windows were drawn from a multivariate Gaussian distribution, with a covariance whose off-diagonal elements are proportional to the overlap between MI estimation windows. Specifically, the multivariate Gaussian sample is a vector with 400 − 250 + 1 = 151 entries, one for each sliding window position within the baseline window. The covariance matrix entry at position *i*,*j* is *σ*^2^ ⋅ (250 − |*i* − *j*|), where *σ*^2^ is the variance of the MI at any point in time and let μ be its expected value. This covariance matrix has 151 × 151 entries. The mean vector of the multivariate Gaussian is 151 entries long, each of which is μ. Estimation of *σ*^2^ and μ is done with closed-form Bayesian inference under a standard Gauss–gamma prior. This procedure results in a Student *t* MI posterior. The significance threshold S was determined by numerical integration of this posterior to the MI value where *p* = 0.05, i.e., we determined S such that

∫S∞dMIΓ(ν+0.5)VΓ(ν)2π(1+dtβ)dt(1+(MI−μdt)2(1+dtβ)dtV2)−ν−0.5=0.05

Where μ, β, ν, and V are the mean, precision, concentration parameter, and shape parameter of the Gauss–gamma posterior obtained by conditioning on the baseline data and dt is the chosen discretization of the time axis (here 1 ms). The code for this algorithm is available from the authors on demand.

Testing for a significant impact of error in trial *n* − 1 on the CS pattern in trial *n* relied on a bootstrapping approach that tried to estimate the size of MI in the primary error period of trial *n* based on a set of trials *n* − 1 without consistent information on error. Hence, we first calculated three MI functions for pairwise comparisons of two sets of trials *n* − 1 (inward error trials versus outward error trials or either of the two versus control) and determined the pair that yielded the largest MI in the period of interest. Next, we calculated the MI for the same trials *n* − 1 after having randomly assigned them to new sets, thereby annihilating the association with a particular condition. This procedure was repeated 500 times for each CS unit and direction, yielding 500 bootstrapped MI functions. We considered a CS unit as exhibiting a significant MI in trial *n* due to the presence of an error in trial *n* − 1 if it exceeded 95% (i.e., the 95th quantile) of these 500 bootstrapped MI functions, provided the MI in the period of interest also deviated significantly from the baseline MI in trial *n*.

We defined the event of alignment for which the MI obtained the largest value relative to baseline as a unit’s event of maximal information. We also determined the interval (Fig 1A) in which the MI peaked. The first interval considered was the primary error interval (II), the time between the target jump and the start of the primary saccade expanded by the following phase of the primary saccade, thereby reaching from the initial target jump to the end of the primary saccade. This is the time during which visual information on target direction and eccentricity guiding the primary saccade, not yet modified by feedback on the saccade, can be processed by the cerebellum. The second interval (III) ranged from the end of the primary saccade to the start of the secondary, corrective saccade. This is the interval in which the information about an erroneous performance becomes available, allowing for the planning and initiation of a corrective saccade. Finally, we considered the post-correction interval (IV) after the corrective saccade in which information about the outcome of the corrective saccade and the overall performance in a given trial is available.

Note that we considered only the maximal significant MI peak independent of error category and alignment and did not take into account lower significant peaks in other conditions or secondary peaks, thus defining exclusively one event per CS unit.

The data underlying all figures and supplemental figures is available from the Dryad Digital Repository: https://doi.org/10.5061/dryad.p88b8v8 [44].

## Supporting information

S1 Fig Two-dimensional view on the paradigm. X, Y-plot of eye position of saccades made in an exemplary experiment. The randomized conditions are distinguished by different colors (control: green; inward error: blue; outward error: red). Underlying data available from the Dryad Digital Repository: https://doi.org/10.5061/dryad.p88b8v8.
(TIF)

S2 Fig Individual contributions of the three monkeys. The bars depict the percentages of significant trial-by-trial adaptation for the various directions studied. Significance was based on ANOVA (see Fig 1 for further details). Underlying data available from the Dryad Digital Repository: https://doi.org/10.5061/dryad.p88b8v8.
(TIF)

S3 Fig Behavioral negative control. Regression analysis for trials from all three monkeys, considering directions with nonsignificant effect of trial-by-trial adaptation. The plot shows a slight deviation from the horizontal in a direction that qualitatively corresponds to the one for significant trials. The slope of 0.021 of this regression line is roughly three times smaller than the one for significant trials shown in Fig 1C and 1D. The confidence interval is 0.018 to 0.024; the effect size 0.16. Underlying data available from the Dryad Digital Repository: https://doi.org/10.5061/dryad.p88b8v8.
(TIF)

S4 Fig Effect of adaptation from trial *n* − 2 to trial *n*. Plot of normalized saccade amplitude in trial *n* as function of normalized visual error in trial *n* − 2 for all directions that had yielded a significant effect of trial *n* − 2 on saccade amplitude in trial *n* as revealed by ANOVA (p < 0.05). The slope of the regression was −0.0047 with a 95% confidence interval at −0.0011 and 0.0015 and a Hedges’ g effect size of 0.028 between the clusters for inward error and outward error trials. In other words, it did not reach significance. Underlying data available from the Dryad Digital Repository: https://doi.org/10.5061/dryad.p88b8v8.
(TIF)

S5 Fig Directional MI analysis for the exemplary unit presented in Fig 2. The presence of direction selectivity was tested for the three different conditions: inward error, outward error, and control. For each condition, the data were aligned to the main four events in a trial (initial target jump, primary saccade start, primary saccade end, corrective saccade start). For each alignment, the individual significance threshold was *p* = 0.05. Note that for this unit, the condition and alignment with inward error and primary saccade end provided the clearest peak and is considered the most informative combination for this CS unit. Underlying data available from the Dryad Digital Repository: https://doi.org/10.5061/dryad.p88b8v8. CS, complex spikes; MI, mutual information.
(TIF)

S6 FigMapping of the point of significant CS modulation.**A** Plot of MI as a function of time for an exemplary CS unit exhibiting its maximal MI after the corrective saccade, when aligned to the onset of the secondary (corrective) saccade. Both panels are based on the same data: The panel on the left shows the MI based on trials aligned to the start of the primary saccade (dashed green line) and the right one to the onset of the corrective saccade (dashed green line). The solid red bar represents the significance threshold as derived from the respective baseline interval (vertical dashed black lines). **B** The top panel depicts the MI as function of time for an exemplary CS unit that underwent the directional preference analysis (i.e., the MI between CS discharge and the eight directions). Trials are aligned to the onset of the primary saccade (green vertical line). The red horizontal line is the individual significance threshold as derived from the baseline interval. The time course from the end of the baseline interval until the end of the trial at 2,200 ms (d’) is mapped onto a normalized time course (d) of a trial as shown in the lower panel. The intervals in the normalized time course were set as follows: a = 200 ms; b = 300 ms; c = 250 ms; d = 1,000 ms. The solid black and the dashed black curve illustrate a schematic eye trace with the corresponding target trace for better orientation. In order to compare the activity across units, we mapped the time point of maximal MI into the normalized time course. This was done in a linear fashion as captured by the ratio $\frac{b\prime }{b+a}=\frac{e\prime }{e}$. By following this procedure for every CS unit, we generated a histogram (grey bars) of the time points of significant CS. The histogram shown is the same as in Fig 2C and based on 79 CS units. Refer to the Results section for further discussion of the analysis and its implications. Underlying data available from the Dryad Digital Repository: https://doi.org/10.5061/dryad.p88b8v8. CS, complex spikes; MI, mutual information.(TIF)

S7 Fig Effect of adaptation for directions with significant MI modulation. Plot of normalized saccade amplitude in trial *n* as function of normalized visual error in trial *n* − 1. All individual saccades (independent of direction) are considered, for which the MI analysis presented in Fig 3C gave significant results (data from *N* = 266 directions). The slope is 0.041 ± 0.004, based on a regression of all three clusters (blue: inward error; green: control; red: outward error). A one-way ANOVA revealed a significant effect of type of target shift (*p* = 1.74 × 10^−117^) and the Hedges’ g as measure of effect size between the clusters of inward and outward shifts amounted to 0.34. Underlying data available from the Dryad Digital Repository: https://doi.org/10.5061/dryad.p88b8v8. MI, mutual information.
(TIF)

S8 Fig Correlation of CS activity in trial *n* and saccade amplitude in trial *n*. Analysis of correlation between the size of the CS activity in the primary error interval and the saccade amplitude in trial *n*, assuming that the CS activity in the period in question will be influenced by the past error. Plots are based on the same datasets as Figs 3C and 4. The plot on the right gives the distribution of correlation coefficients obtained when pooling all cases. The distribution is centered on zero with a tiny yet significant preponderance of negative correlations. The left panel distinguishes two distributions considering the two possible alignments of a unit’s preferred direction with the direction of the visual error (see Fig 4 for additional information; the distribution for antiparallel orientations is plotted in blue, the one for parallel orientations in red; the red distribution is not different from zero [*t* test, *p* = 0.84; effect size: 0.017], whereas the blue population lies in the negative area, indicating that inward errors lead to a higher number of CS [*p* = 0.0054; effect size: 0.25]). Both populations differ significantly from one another (*t* test, *p* < 0.05; effect size: 0.29). Underlying data available from the Dryad Digital Repository: https://doi.org/10.5061/dryad.p88b8v8. CS, complex spikes.
(TIF)

S9 Fig Significant SS modulation. Distribution of time points of significant SS modulation analogous to Fig 3C for cases in which a significant effect of past errors on the CS modulation had been obtained. The red curve of “estimated probability” is, as in the population plots of Figs 2C & 3C, a kernel density estimation for illustration purposes only. We obtained a significant SS modulation prior to the primary saccade end for 104 directions, which are 19.7% of the 526 directions. Considering SS for which the corresponding CS also showed a significant modulation, the values change to 35 directions out of 193, which are 18.1%. Underlying data available from the Dryad Digital Repository: https://doi.org/10.5061/dryad.p88b8v8. CS, complex spikes; SS, simple spikes.
(TIF)

S10 Fig CS recognition is guided by its canonical shape and LFP signal. **A** Exemplary PC recording showing a typical complex spike (CS, asterisk) followed by three SS. The upper trace shows the 250 Hz–10 kHz band pass–filtered signal, whereas the lower trace depicts the low pass–filtered LFP signal with a cutoff frequency of 150 Hz. Note the long-lasting upward deflection in the LFP caused by the polyphasic complex spike. **B** Example of CS observed in isolation from SS, arguably recorded further away from the cell body in the molecular layer (upper panel). Again, the lower trace shows a marked LFP deflection paralleling the occurrence of the CS. **C** Example of a less-well–isolated PC, exhibiting SS framed by two CS at 0 and 55 ms. The offline detection of CS in this and similar recordings was greatly facilitated by considering the LFP signal (lower trace). **D** Plot of the number of spikelets per CS unit as function of its duration; the latter occurs binned by the discrete increase in duration with each additional spikelet. The plot shows data from one CS unit and the inlay depicts an exemplary CS waveform with three spikelets. Underlying data available from the Dryad Digital Repository: https://doi.org/10.5061/dryad.p88b8v8. CS, complex spikes; LFP, local field potential; PC, Purkinje cell; SS, simple spikes.
(TIF)

S11 Fig Effects of CS duration in the random error paradigm. **A** Distributions of mean CS duration for the short (2 ms to 7 ms), the medium (3 ms to 8 ms), and the long (3 ms to 12 ms) CS durations based on *N* = 38 CS. The calculation for the binning is described in S1 Text. **B** Box and whisker plot of the average CS duration for the four trial periods distinguished given that ≤ 2 CS occurred in the respective interval. The boxes give the median and the whiskers indicate the 95th-percentile range. A Kruskal–Wallis-test showed no significant difference between the four trial periods (*p* > 0.05). **C** Plot of duration of CS fired during baseline fixation as function of duration of the same CS when generated in the presence of a secondary visual error (*N* = 34 CS units considered). The dashed line is the identity line; the solid line is a regression line fitted to the data points. It has a slope of 0.73 and an intercept of 1.31 ms. **D** Box and whisker plot (format as in B) of change of saccade amplitude from trial *n* − 1 to trial *n* as function of CS duration in the secondary visual error interval (III in Fig 1B) in trial *n* − 1 (3 bins: short, medium, or long CS duration). The data point on the left depicts the change in the absence of a CS; ≤ 2 CS were required in the respective interval for a CS unit to be considered. Underlying data available from the Dryad Digital Repository: https://doi.org/10.5061/dryad.p88b8v8. CS, complex spikes.
(TIF)

S12 Fig Correlation of visual error in trial *n* − 1 and SS activity in trial *n*. SS density functions analogous to Fig 4D. The SS SDF are computed out of those trials for which the CS modulation exhibits a positive correlation coefficient and for which the average SS rate of the respective SS unit remained stable between 30Hz and 60Hz to avoid bias. The red trace indicates an outward error on trial *n*– 1, and the blue trace those trials with an inward error in trial *n* − 1. The grey trace depicts those trials for which no induced visual error was present in trial *n* − 1. The vertical dashed lines represent (from left to right) the initial target shift, the start of the primary saccade, and the end of the primary saccade. The grey curve on the bottom depicts the effect size between the red and the blue curve and times of significant differences (*t* test, *p* < 0.05) are indicated in green. Note that a significant difference between inward and outward error conditions in trial *n* − 1 becomes visible shortly before the primary saccade or around the primary saccade, depending on the correlation coefficient of the CS rate of the very same PC. The solid curve in the center of each panel is an exemplary eye trace with a primary saccade of 10-degree amplitude as in Fig 4D. Underlying data available from the Dryad Digital Repository: https://doi.org/10.5061/dryad.p88b8v8. CS, complex spikes; SDF, spike density function; SS, simple spikes.
(TIF)

S1 Table Contributions to the directional analysis. Overview of results of MI-based CS directionality analysis composing the histogram in Fig 2C. The population of CS units considered for analysis comprised *N* = 129 units. Out of this group, *N* = 79 (61.2%) exhibited significant MI values in any of the periods considered. “Error Condition” distinguishes significant MI values in the three categories of inward error, outward error, or no error control. “Time point of alignment” are the time points of the oculomotor events to which spike trains were aligned prior to MI analysis. “Interval” distinguishes the three longer-lasting trial periods following the baseline period. “Two significant MI peaks” indicates units whose MI time course crossed the significance threshold twice for the same alignment. Percentages are rounded to one-tenth of a percent. CS, complex spikes; MI, mutual information.
(DOCX)

S1 Text Analysis of CS duration. A description of the analysis and the results of the analysis of the CS duration. CS, complex spikes.
(DOCX)

## Abbreviations

CS: complex spikes
LFP: local field potential
MAI: Marr–Albus–Ito
MI: mutual information
NHP: nonhuman primate
OMV: oculomotor vermis
PC: Purkinje cell
PSTH: peri stimulus time histogram
SC: superior colliculus
SDF: spike density function
SNR: signal-to-noise ratio
SS: simple spikes
STSA: short-term saccadic adaptation
