# Supplementary figures and images for: Learning from the past: A reverberation of past errors in the cerebellar climbing fiber signal
Source: PLoS Biol. 2018 Aug 1;16(8):e2004344. doi: 10.1371/journal.pbio.2004344 (PMC6089447; doi:10.1371/journal.pbio.2004344)

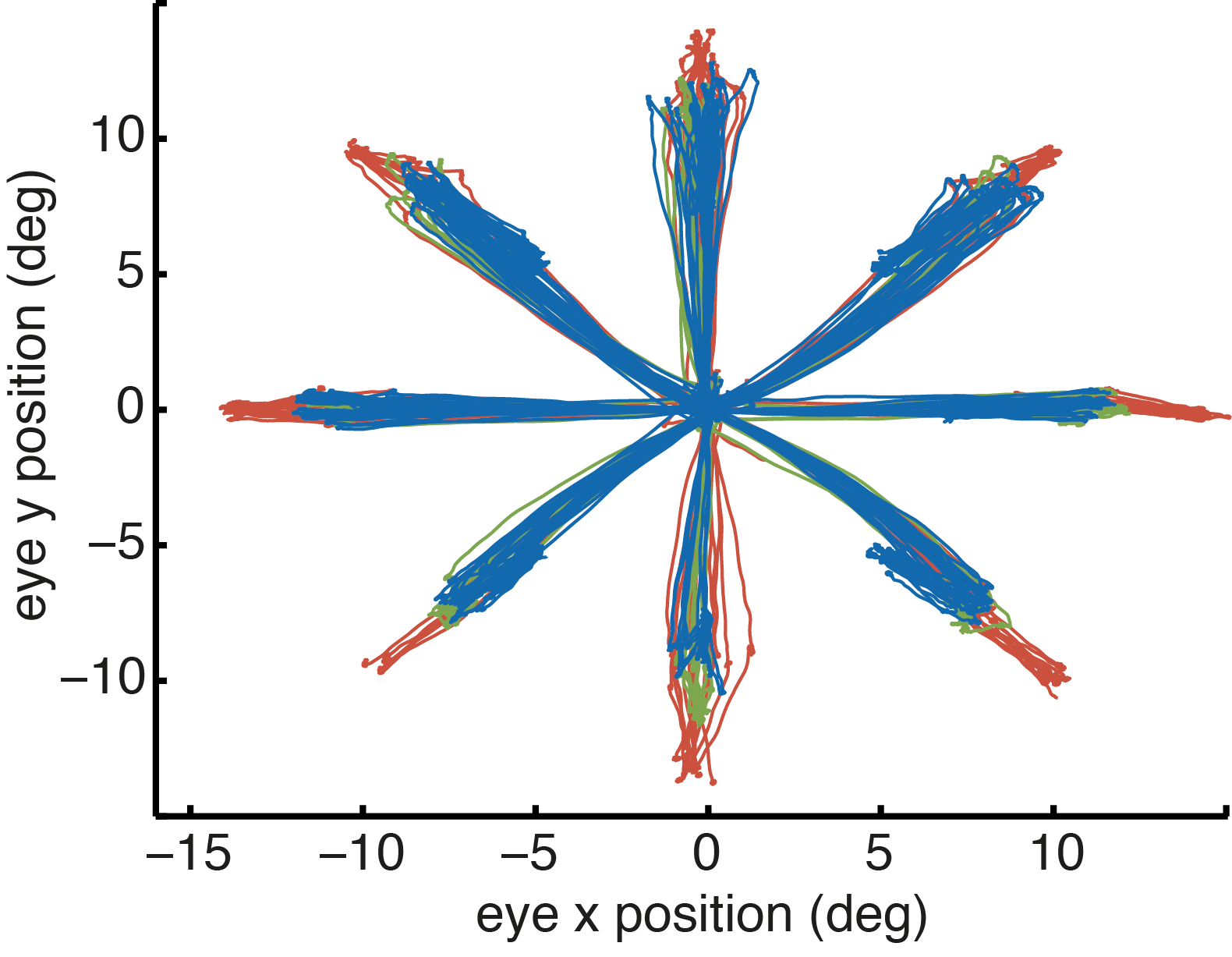

Supplement: S1 Fig — X, Y-plot of eye position of saccades made in an exemplary experiment. The randomized conditions are distinguished by different colors (control: green; inward error: blue; outward error: red). Underlying data available from the Dryad Digital Repository: https://doi.org/10.5061/dryad.p88b8v8. (TIF) [file pbio.2004344.s001.tif]

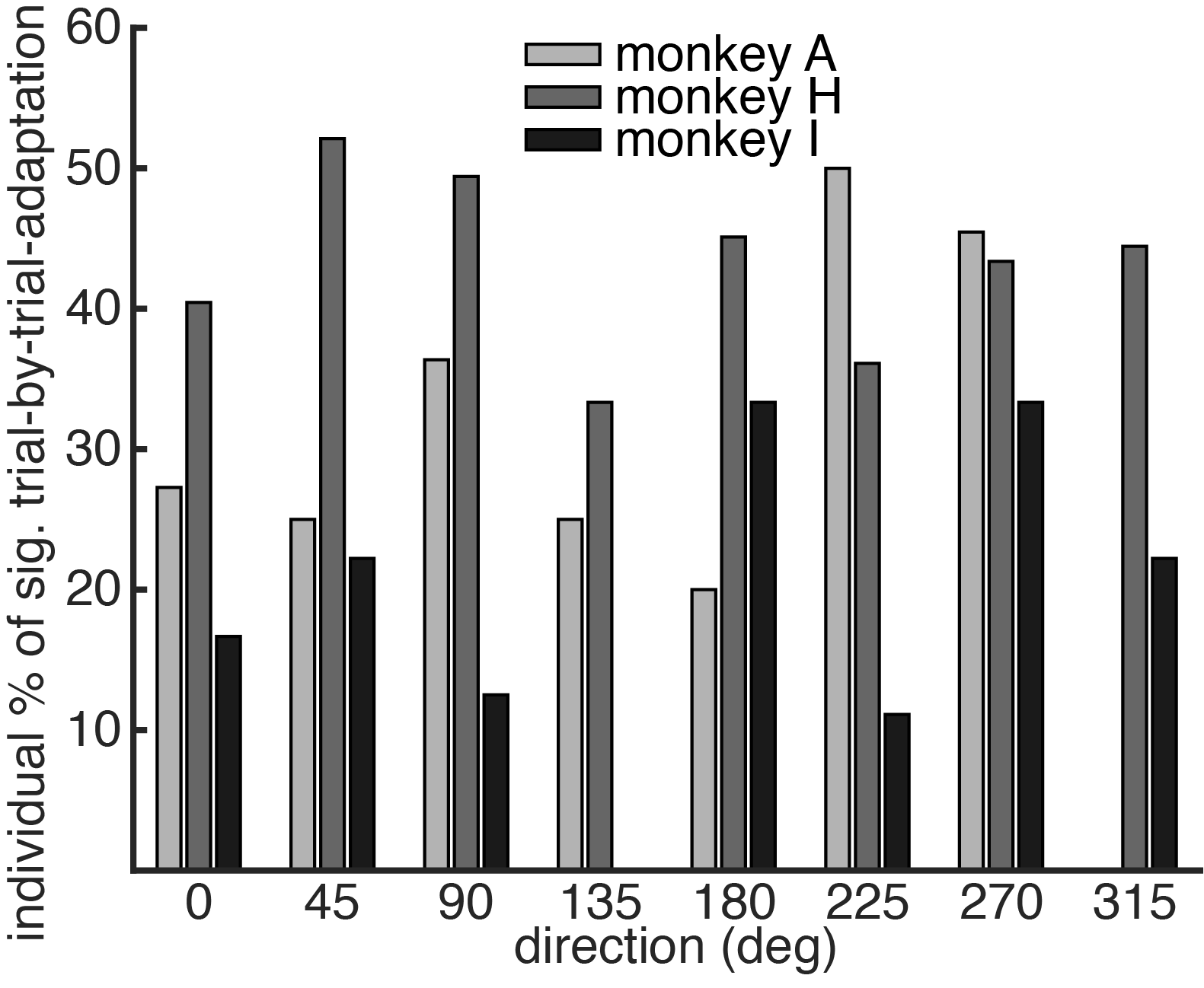

Supplement: S2 Fig — The bars depict the percentages of significant trial-by-trial adaptation for the various directions studied. Significance was based on ANOVA (see Fig 1 for further details). Underlying data available from the Dryad Digital Repository: https://doi.org/10.5061/dryad.p88b8v8. (TIF) [file pbio.2004344.s002.tif]

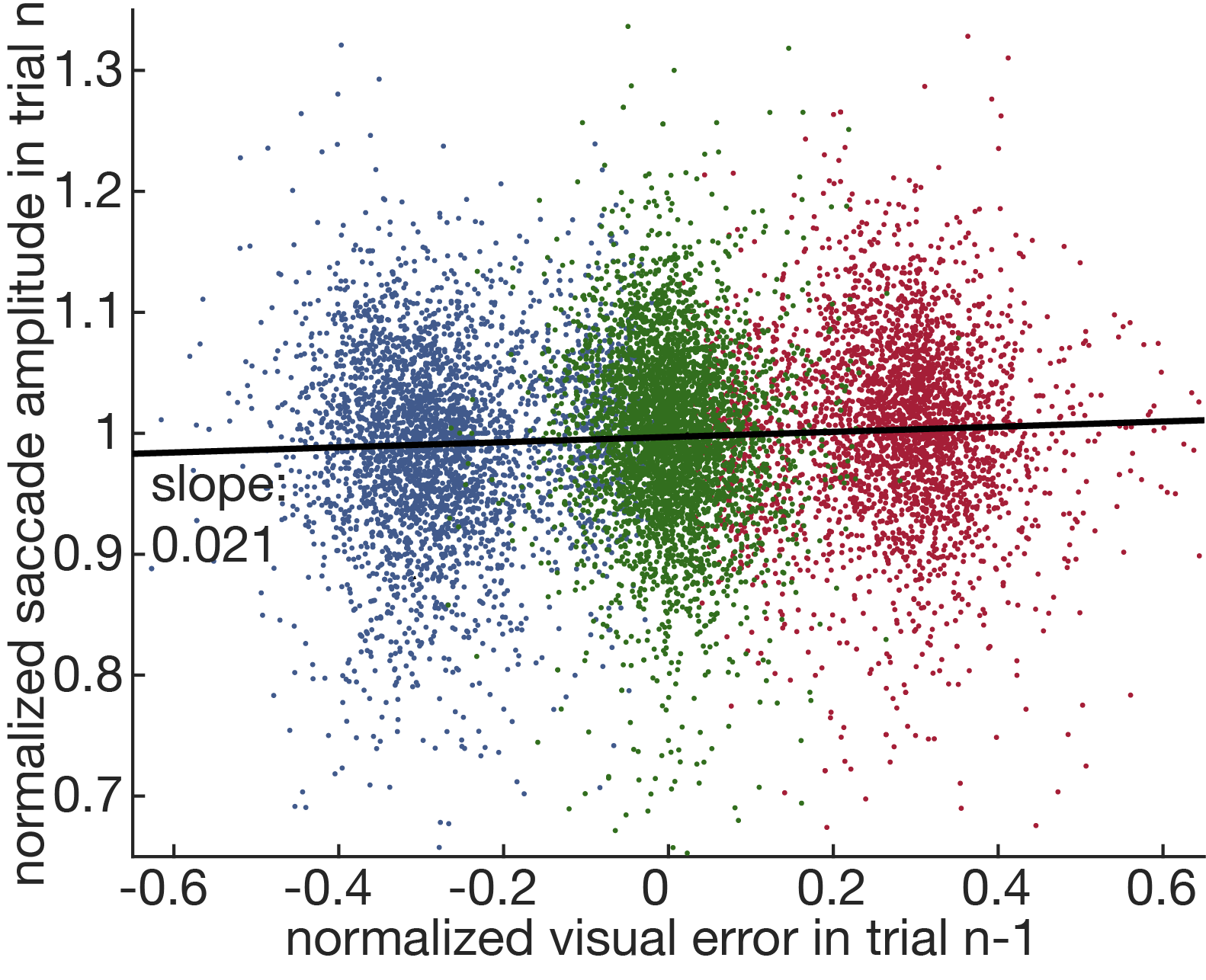

Supplement: S3 Fig — Regression analysis for trials from all three monkeys, considering directions with nonsignificant effect of trial-by-trial adaptation. The plot shows a slight deviation from the horizontal in a direction that qualitatively corresponds to the one for significant trials. The slope of 0.021 of this regression line is roughly three times smaller than the one for significant trials shown in Fig 1C and 1D. The confidence interval is 0.018 to 0.024; the effect size 0.16. Underlying data available from the Dryad Digital Repository: https://doi.org/10.5061/dryad.p88b8v8. (TIF) [file pbio.2004344.s003.tif]

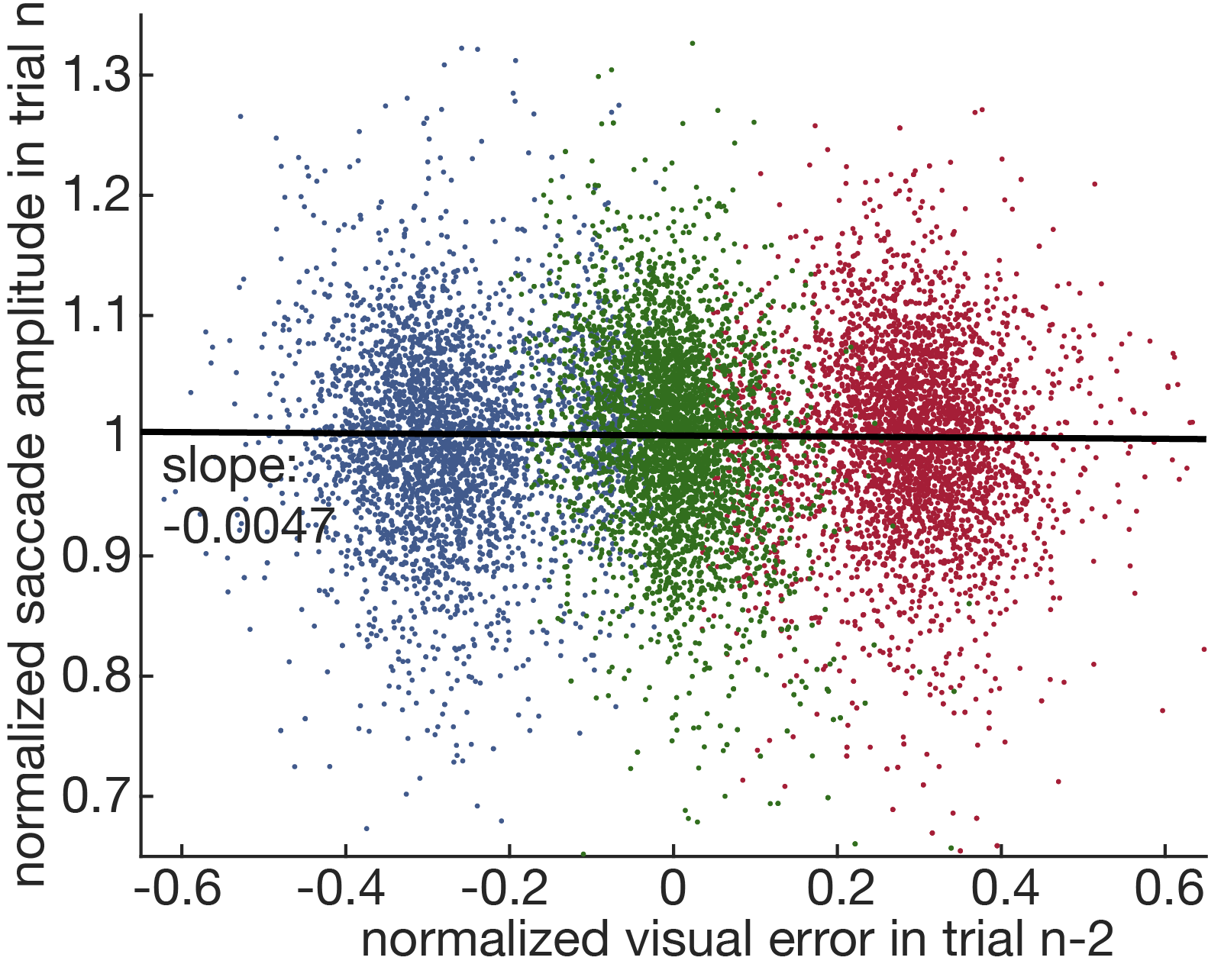

Supplement: S4 Fig — Plot of normalized saccade amplitude in trial n as function of normalized visual error in trial n − 2 for all directions that had yielded a significant effect of trial n − 2 on saccade amplitude in trial n as revealed by ANOVA (p < 0.05). The slope of the regression was −0.0047 with a 95% confidence interval at −0.0011 and 0.0015 and a Hedges’ g effect size of 0.028 between the clusters for inward error and outward error trials. In other words, it did not reach significance. Underlying data available from the Dryad Digital Repository: https://doi.org/10.5061/dryad.p88b8v8. (TIF) [file pbio.2004344.s004.tif]

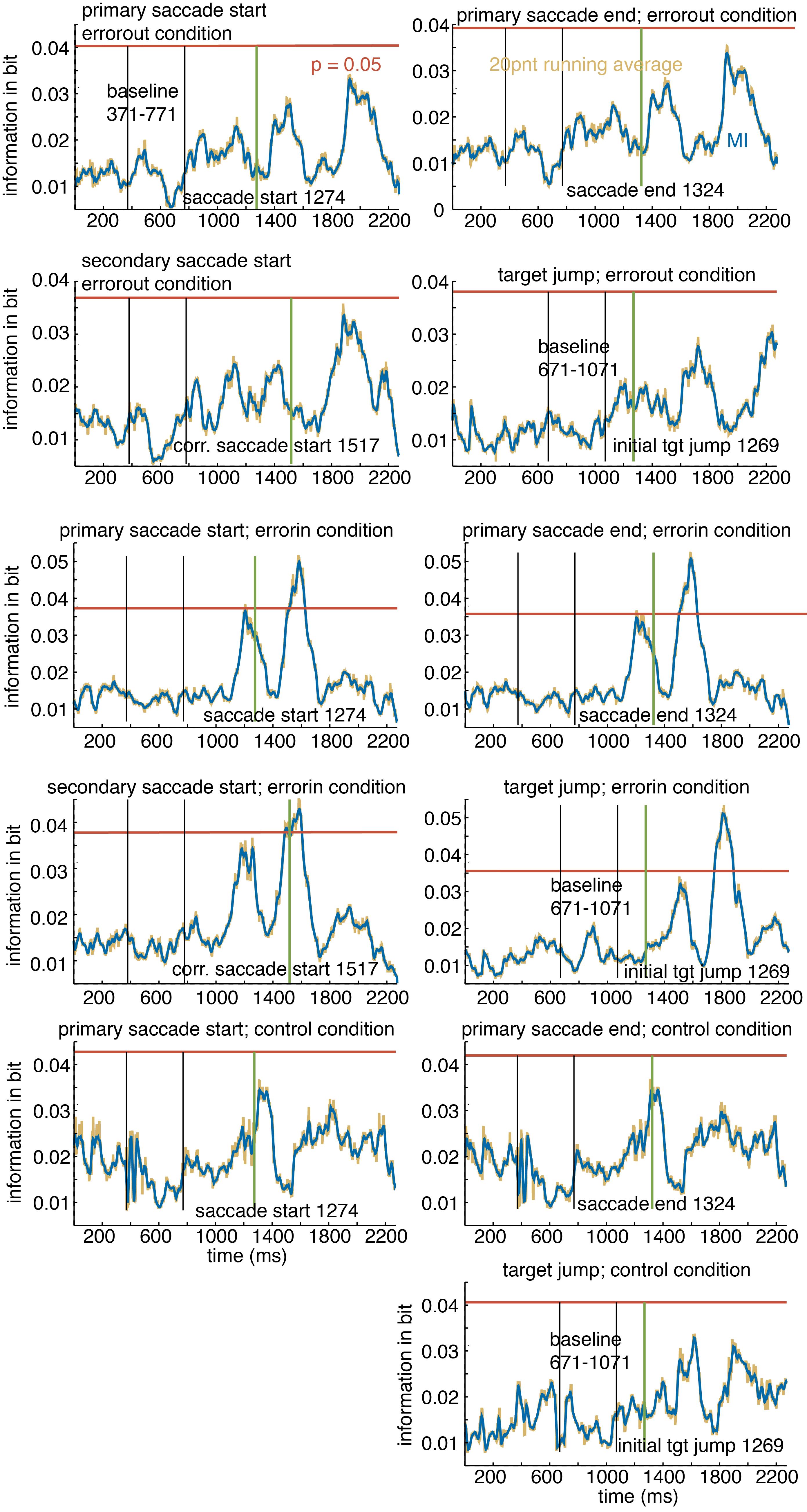

Supplement: S5 Fig — The presence of direction selectivity was tested for the three different conditions: inward error, outward error, and control. For each condition, the data were aligned to the main four events in a trial (initial target jump, primary saccade start, primary saccade end, corrective saccade start). For each alignment, the individual significance threshold was p = 0.05. Note that for this unit, the condition and alignment with inward error and primary saccade end provided the clearest peak and is considered the most informative combination for this CS unit. Underlying data available from the Dryad Digital Repository: https://doi.org/10.5061/dryad.p88b8v8. CS, complex spikes; MI, mutual information. (TIF) [file pbio.2004344.s005.tif]

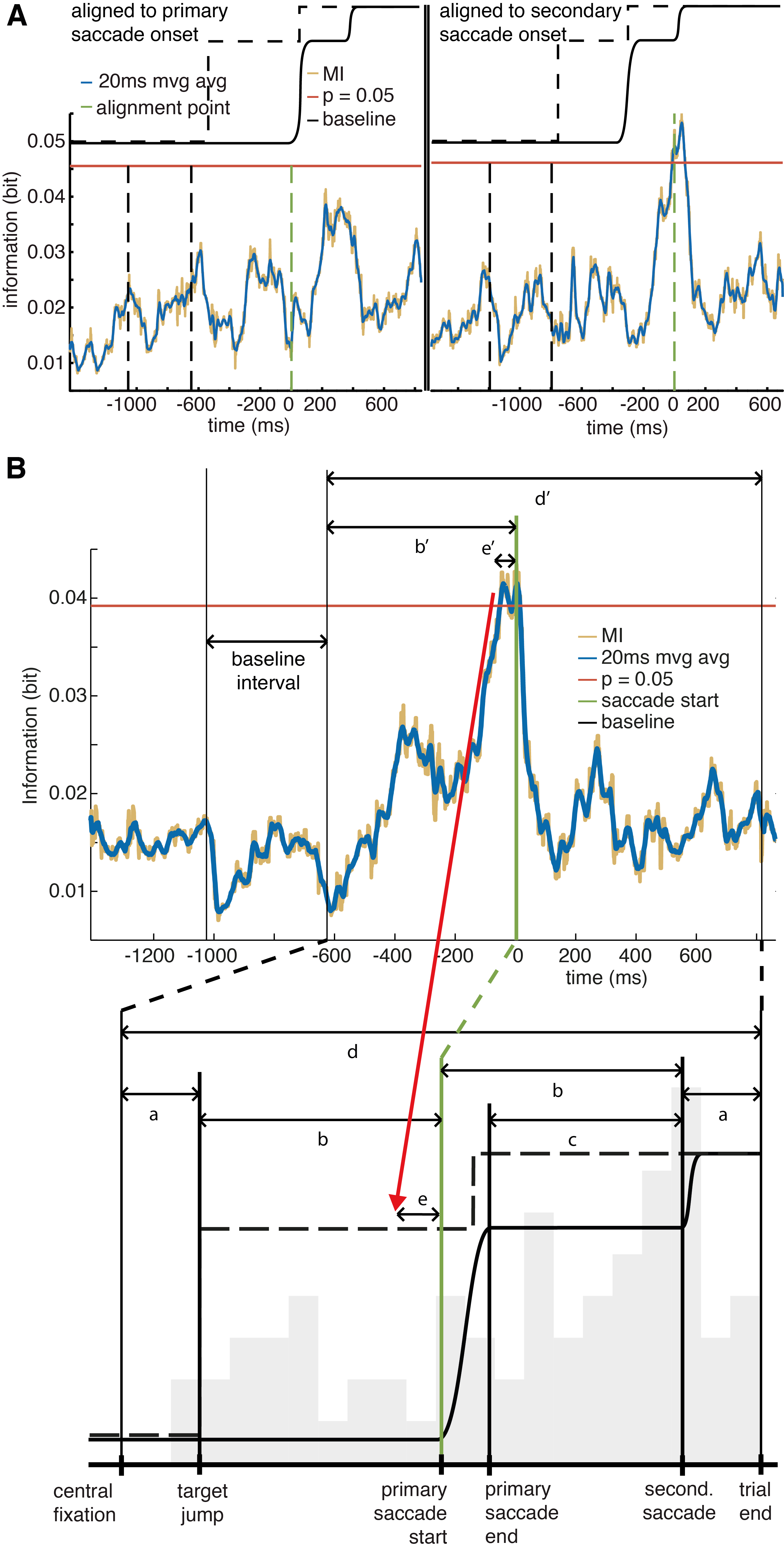

Supplement: S6 Fig — A Plot of MI as a function of time for an exemplary CS unit exhibiting its maximal MI after the corrective saccade, when aligned to the onset of the secondary (corrective) saccade. Both panels are based on the same data: The panel on the left shows the MI based on trials aligned to the start of the primary saccade (dashed green line) and the right one to the onset of the corrective saccade (dashed green line). The solid red bar represents the significance threshold as derived from the respective baseline interval (vertical dashed black lines). B The top panel depicts the MI as function of time for an exemplary CS unit that underwent the directional preference analysis (i.e., the MI between CS discharge and the eight directions). Trials are aligned to the onset of the primary saccade (green vertical line). The red horizontal line is the individual significance threshold as derived from the baseline interval. The time course from the end of the baseline interval until the end of the trial at 2,200 ms (d’) is mapped onto a normalized time course (d) of a trial as shown in the lower panel. The intervals in the normalized time course were set as follows: a = 200 ms; b = 300 ms; c = 250 ms; d = 1,000 ms. The solid black and the dashed black curve illustrate a schematic eye trace with the corresponding target trace for better orientation. In order to compare the activity across units, we mapped the time point of maximal MI into the normalized time course. This was done in a linear fashion as captured by the ratio b′b+a=e′e. By following this procedure for every CS unit, we generated a histogram (grey bars) of the time points of significant CS. The histogram shown is the same as in Fig 2C and based on 79 CS units. Refer to the Results section for further discussion of the analysis and its implications. Underlying data available from the Dryad Digital Repository: https://doi.org/10.5061/dryad.p88b8v8. CS, complex spikes; MI, mutual information. (TIF) [file pbio.2004344.s006.tif]

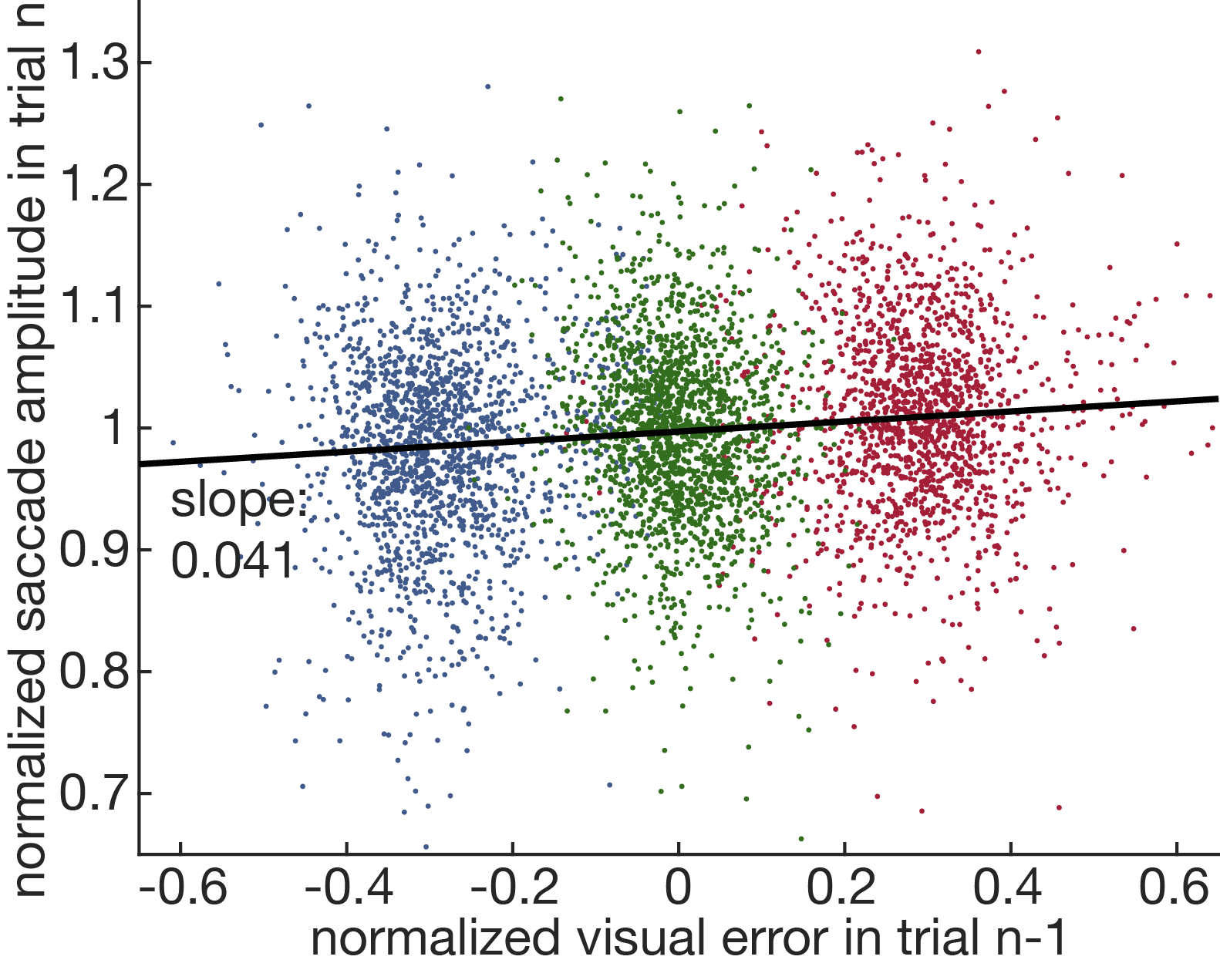

Supplement: S7 Fig — Plot of normalized saccade amplitude in trial n as function of normalized visual error in trial n − 1. All individual saccades (independent of direction) are considered, for which the MI analysis presented in Fig 3C gave significant results (data from N = 266 directions). The slope is 0.041 ± 0.004, based on a regression of all three clusters (blue: inward error; green: control; red: outward error). A one-way ANOVA revealed a significant effect of type of target shift (p = 1.74 × 10−117) and the Hedges’ g as measure of effect size between the clusters of inward and outward shifts amounted to 0.34. Underlying data available from the Dryad Digital Repository: https://doi.org/10.5061/dryad.p88b8v8. MI, mutual information. (TIF) [file pbio.2004344.s007.tif]

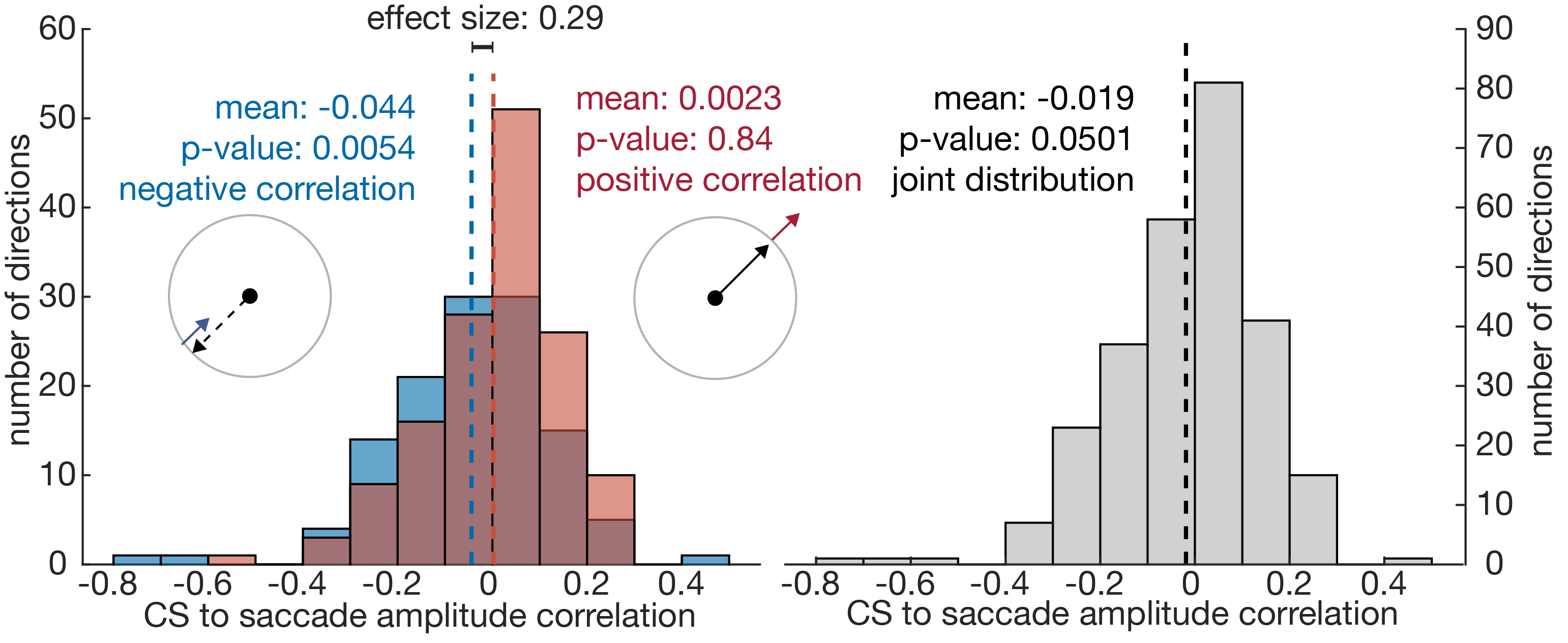

Supplement: S8 Fig — Analysis of correlation between the size of the CS activity in the primary error interval and the saccade amplitude in trial n, assuming that the CS activity in the period in question will be influenced by the past error. Plots are based on the same datasets as Figs 3C and 4. The plot on the right gives the distribution of correlation coefficients obtained when pooling all cases. The distribution is centered on zero with a tiny yet significant preponderance of negative correlations. The left panel distinguishes two distributions considering the two possible alignments of a unit’s preferred direction with the direction of the visual error (see Fig 4 for additional information; the distribution for antiparallel orientations is plotted in blue, the one for parallel orientations in red; the red distribution is not different from zero [t test, p = 0.84; effect size: 0.017], whereas the blue population lies in the negative area, indicating that inward errors lead to a higher number of CS [p = 0.0054; effect size: 0.25]). Both populations differ significantly from one another (t test, p < 0.05; effect size: 0.29). Underlying data available from the Dryad Digital Repository: https://doi.org/10.5061/dryad.p88b8v8. CS, complex spikes. (TIF) [file pbio.2004344.s008.tif]

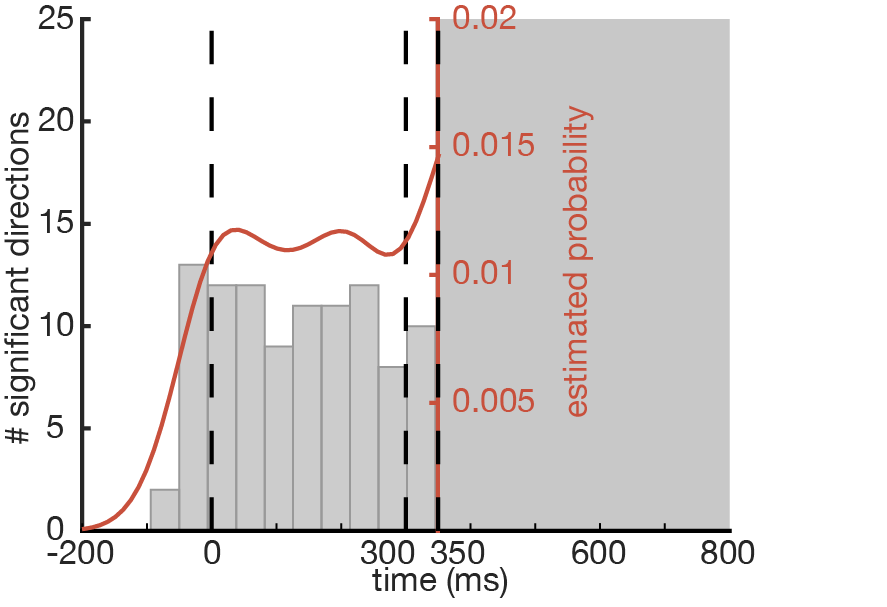

Supplement: S9 Fig — Distribution of time points of significant SS modulation analogous to Fig 3C for cases in which a significant effect of past errors on the CS modulation had been obtained. The red curve of “estimated probability” is, as in the population plots of Figs 2C & 3C, a kernel density estimation for illustration purposes only. We obtained a significant SS modulation prior to the primary saccade end for 104 directions, which are 19.7% of the 526 directions. Considering SS for which the corresponding CS also showed a significant modulation, the values change to 35 directions out of 193, which are 18.1%. Underlying data available from the Dryad Digital Repository: https://doi.org/10.5061/dryad.p88b8v8. CS, complex spikes; SS, simple spikes. (TIF) [file pbio.2004344.s009.tif]

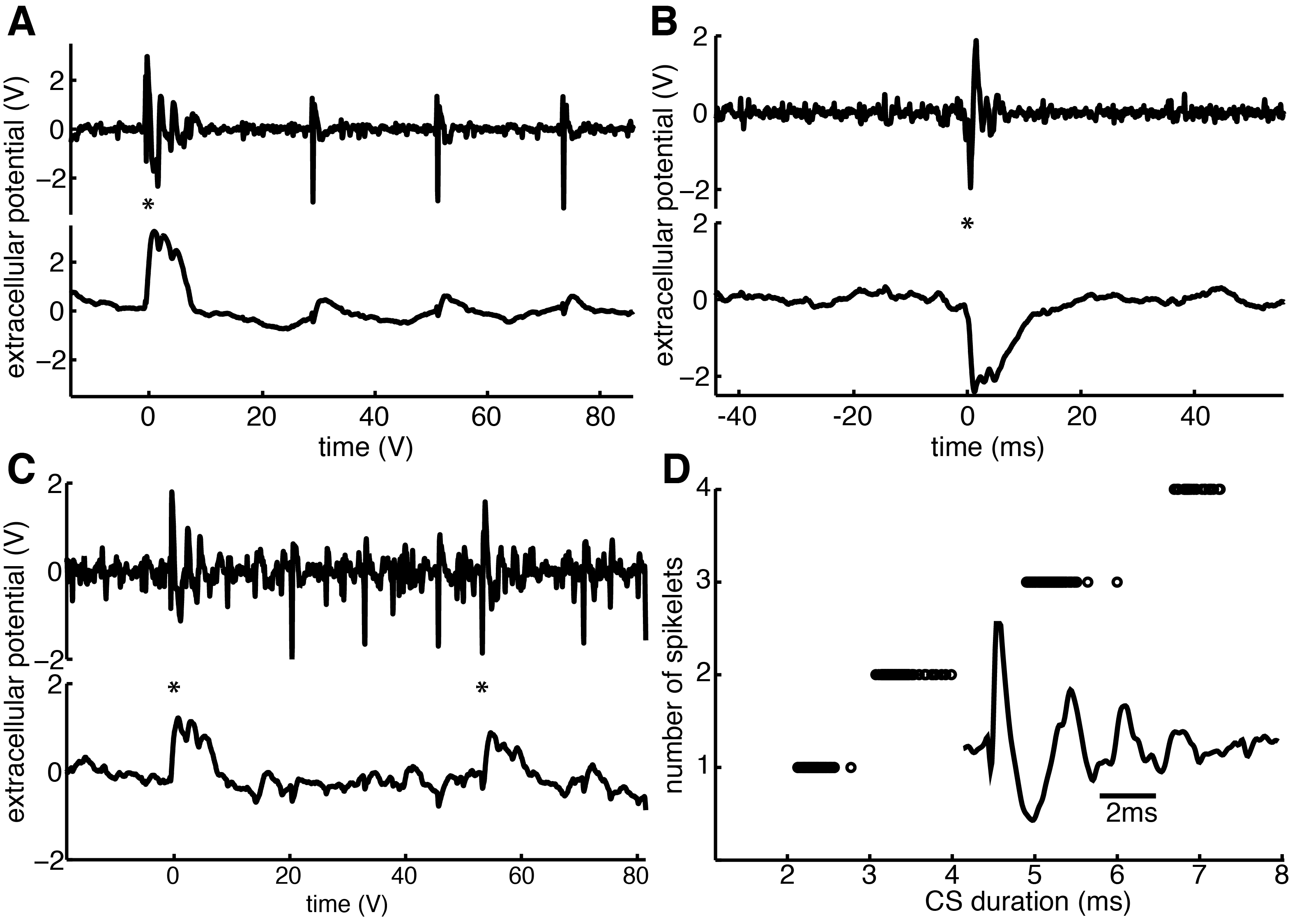

Supplement: S10 Fig — A Exemplary PC recording showing a typical complex spike (CS, asterisk) followed by three SS. The upper trace shows the 250 Hz–10 kHz band pass–filtered signal, whereas the lower trace depicts the low pass–filtered LFP signal with a cutoff frequency of 150 Hz. Note the long-lasting upward deflection in the LFP caused by the polyphasic complex spike. B Example of CS observed in isolation from SS, arguably recorded further away from the cell body in the molecular layer (upper panel). Again, the lower trace shows a marked LFP deflection paralleling the occurrence of the CS. C Example of a less-well–isolated PC, exhibiting SS framed by two CS at 0 and 55 ms. The offline detection of CS in this and similar recordings was greatly facilitated by considering the LFP signal (lower trace). D Plot of the number of spikelets per CS unit as function of its duration; the latter occurs binned by the discrete increase in duration with each additional spikelet. The plot shows data from one CS unit and the inlay depicts an exemplary CS waveform with three spikelets. Underlying data available from the Dryad Digital Repository: https://doi.org/10.5061/dryad.p88b8v8. CS, complex spikes; LFP, local field potential; PC, Purkinje cell; SS, simple spikes. (TIF) [file pbio.2004344.s010.tif]

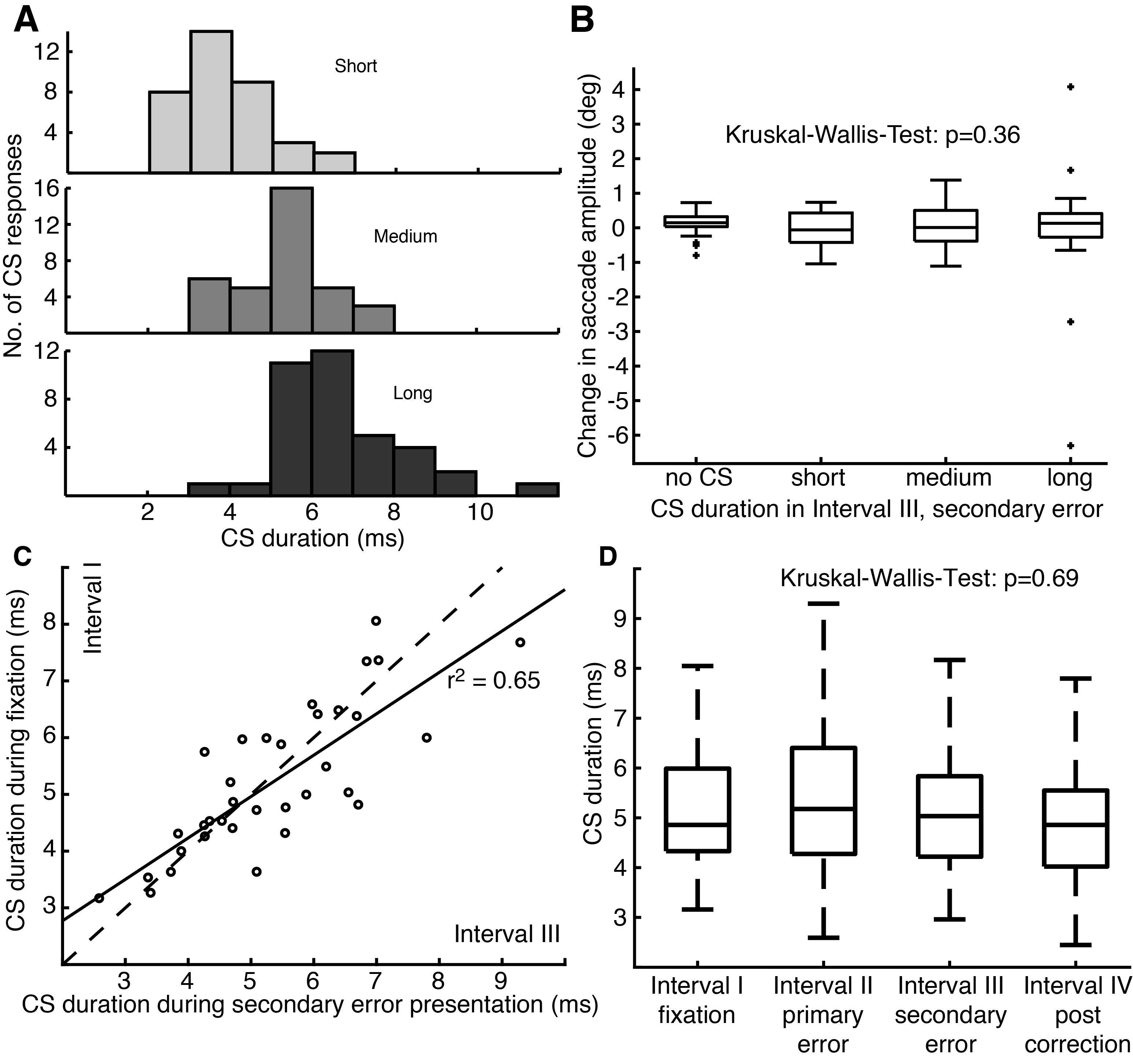

Supplement: S11 Fig — A Distributions of mean CS duration for the short (2 ms to 7 ms), the medium (3 ms to 8 ms), and the long (3 ms to 12 ms) CS durations based on N = 38 CS. The calculation for the binning is described in S1 Text. B Box and whisker plot of the average CS duration for the four trial periods distinguished given that ≤ 2 CS occurred in the respective interval. The boxes give the median and the whiskers indicate the 95th-percentile range. A Kruskal–Wallis-test showed no significant difference between the four trial periods (p > 0.05). C Plot of duration of CS fired during baseline fixation as function of duration of the same CS when generated in the presence of a secondary visual error (N = 34 CS units considered). The dashed line is the identity line; the solid line is a regression line fitted to the data points. It has a slope of 0.73 and an intercept of 1.31 ms. D Box and whisker plot (format as in B) of change of saccade amplitude from trial n − 1 to trial n as function of CS duration in the secondary visual error interval (III in Fig 1B) in trial n − 1 (3 bins: short, medium, or long CS duration). The data point on the left depicts the change in the absence of a CS; ≤ 2 CS were required in the respective interval for a CS unit to be considered. Underlying data available from the Dryad Digital Repository: https://doi.org/10.5061/dryad.p88b8v8. CS, complex spikes. (TIF) [file pbio.2004344.s011.tif]

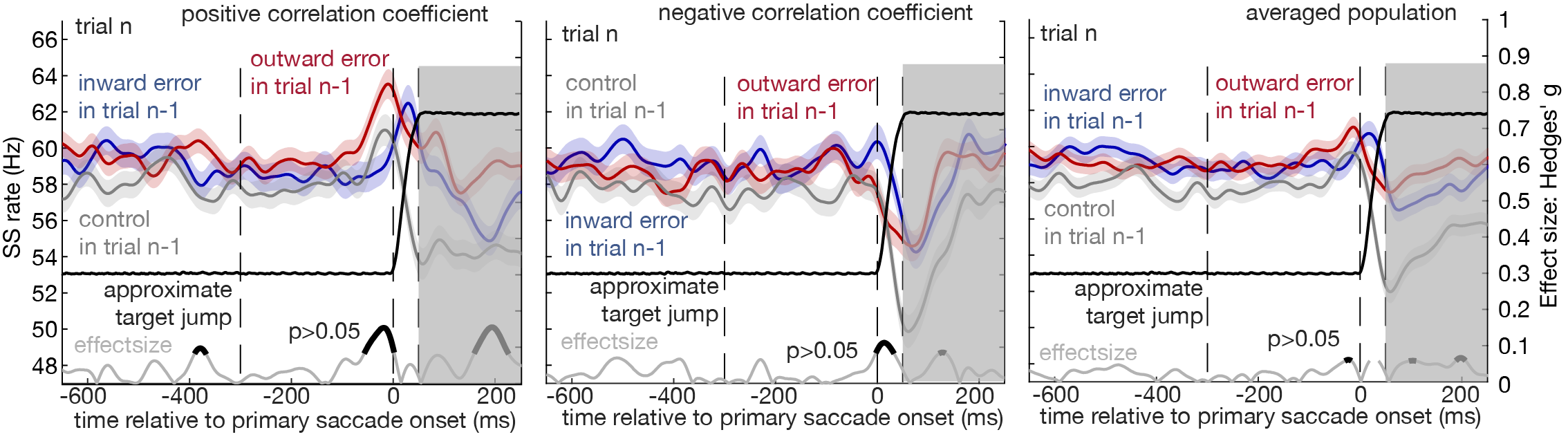

Supplement: S12 Fig — SS density functions analogous to Fig 4D. The SS SDF are computed out of those trials for which the CS modulation exhibits a positive correlation coefficient and for which the average SS rate of the respective SS unit remained stable between 30Hz and 60Hz to avoid bias. The red trace indicates an outward error on trial n– 1, and the blue trace those trials with an inward error in trial n − 1. The grey trace depicts those trials for which no induced visual error was present in trial n − 1. The vertical dashed lines represent (from left to right) the initial target shift, the start of the primary saccade, and the end of the primary saccade. The grey curve on the bottom depicts the effect size between the red and the blue curve and times of significant differences (t test, p < 0.05) are indicated in green. Note that a significant difference between inward and outward error conditions in trial n − 1 becomes visible shortly before the primary saccade or around the primary saccade, depending on the correlation coefficient of the CS rate of the very same PC. The solid curve in the center of each panel is an exemplary eye trace with a primary saccade of 10-degree amplitude as in Fig 4D. Underlying data available from the Dryad Digital Repository: https://doi.org/10.5061/dryad.p88b8v8. CS, complex spikes; SDF, spike density function; SS, simple spikes. (TIF) [file pbio.2004344.s012.tif]
