# Supplementary material for: Learning from the past: A reverberation of past errors in the cerebellar climbing fiber signal
Source: PLoS Biol. 2018 Aug 1;16(8):e2004344. doi: 10.1371/journal.pbio.2004344 (PMC6089447; doi:10.1371/journal.pbio.2004344)
Supplement: S1 Table — Overview of results of MI-based CS directionality analysis composing the histogram in Fig 2C. The population of CS units considered for analysis comprised N = 129 units. Out of this group, N = 79 (61.2%) exhibited significant MI values in any of the periods considered. “Error Condition” distinguishes significant MI values in the three categories of inward error, outward error, or no error control. “Time point of alignment” are the time points of the oculomotor events to which spike trains were aligned prior to MI analysis. “Interval” distinguishes the three longer-lasting trial periods following the baseline period. “Two significant MI peaks” indicates units whose MI time course crossed the significance threshold twice for the same alignment. Percentages are rounded to one-tenth of a percent. CS, complex spikes; MI, mutual information. (DOCX) [file pbio.2004344.s013.docx]

| **Error condition** | # of significant modulating units | Percentage of population (n=129) | Percentage of significant modulating units (n=79) |
| --- | --- | --- | --- |
| Control | 13 | 10.1% | 16.5% |
| Inward error | 27 | 20.9% | 34.2% |
| Outward error | 39 | 30.2% | 49.4% |
| **Time point of alignment** | # of significant modulating units | Percentage of population | Percentage of significant modulating units |
| Target jump | 15 | 11.6% | 19% |
| Primary saccade start | 20 | 15.5% | 25.3% |
| Primary saccade end | 15 | 11.6% | 19% |
| Corrective saccade start | 29 | 22.5% | 36.7% |
| **Interval** | # of significant modulating units | Percentage of population | Percentage of significant modulating units |
| Primary error interval (II) | 29 | 22.5% | 36.7% |
| Secondary error interval (III) | 34 | 26.4% | 43% |
| Post correction interval (IV) | 16 | 12.4% | 20.3% |
| Two significant MI peaks | 23 | 17.8% | 29.1% |
