# Supplementary material for: Learning from the past: A reverberation of past errors in the cerebellar climbing fiber signal
Source: PLoS Biol. 2018 Aug 1;16(8):e2004344. doi: 10.1371/journal.pbio.2004344 (PMC6089447; doi:10.1371/journal.pbio.2004344)
Supplement: S1 Text — A description of the analysis and the results of the analysis of the CS duration. CS, complex spikes. (DOCX) [file pbio.2004344.s014.docx]

**Complex spike duration does not change in conjunction with trial-by-trial adaptation**

We were able to clearly determine the CS duration in a subset of 38 units of the 129 CS units recorded. Following previous work [9,16] we first determined for each unit the preferred direction (see Materials and methods). For the subsequent analysis of possible adaptation effects on CS duration, only trials that fell into the preferred direction were considered. For four of the 38 units considered, the number of CS in the error interval was too small ($\leq2$) to arrive at reasonable estimates of CS duration. In the remaining 34 units we determined the mean CS duration in the secondary error interval (III) and plotted mean CS duration in the fixation interval (interval I, Fig 1B) as function of the former. There was no systematic difference between the two as indicated by the fact that the linear regression showed no systematic offset with respect to the identity line (S11C Fig). Actually, in none of the intervals considered did the CS duration differ significantly from the one in the fixation interval (S11D Fig).

We next asked if the CS duration in the error interval of trial *n* - 1 had an influence on saccade amplitude in the subsequent trial *n*. To this end, we distinguished four different classes of trials *n* - 1, first, the subset of trials without any CS in the secondary error interval (III) and second, the three CS duration classes, representing „short“, „medium“ and „long“ CS duration in the secondary error interval (III) of trial *n* - 1 trials (S11A Fig; see Materials and methods for details). We then determined the impact of CS class in trial *n* - 1 on the amplitude of the primary saccade in subsequent trial *n* by calculating the change of primary saccade amplitude from trial *n* - 1 to trial *n.* Remember that only trials performed into the preferred direction of that cell with an outward error condition were used as error trials (trial *n* - 1*)* and that trial *n* is the next trial performed into that same direction. As shown in S11B Fig the change in saccade amplitude did not vary significantly with CS class. In other words, the trial-by-trial adaptation elicited by the random error paradigm deployed in our study was independent of changes in CS duration in the secondary error interval (III).

A sampling bias as cause of our failure to unravel a role of duration changes can be excluded, as the 38 CS units subjected to the analysis above do not differ from the complete sample in terms of directional preferences and an influence of the error (29 out of 38 units, 76.3%, show a significant impact either of the error in the directional MI or in the trial-by-trial MI analysis; compared to 88.3% of the full population).
